# Effects of a vegetarian diet combined with aerobic exercise on glycemic control, insulin resistance, and body composition: a systematic review and meta-analysis

**DOI:** 10.1007/s40519-023-01536-5

**Published:** 2023-02-15

**Authors:** Yi Long, Hua Ye, Jiaming Yang, Xi Tao, Huiyong Xie, Jiahong Zhang, Yanbiao Zhong, Maoyuan Wang

**Affiliations:** 1 The First Affiliated Hospital of Gannan Medical University, Ganzhou, Jiangxi Province China; 2 Gannan Medical University, Ganzhou, Jiangxi Province China; 3 Ganzhou Intelligent Rehabilitation Technology Innovation Center, Ganzhou, Jiangxi Province China; 4 Ganzhou Key Laboratory of Rehabilitation Medicine, Ganzhou, Jiangxi Province China

**Keywords:** Vegetarian diet, Aerobic exercise, Body composition, Insulin resistance, Blood glucose

## Abstract

**Background:**

Vegetarian diets and aerobic exercise are increasingly accepted as a common way to improve lifestyle. Several studies have shown that vegetarian diets combined with aerobic exercise interventions have a significant effect on preventing and reducing the risk of metabolic diseases.

**Methods:**

A search of the PubMed, EBSCO, Embase, CENTRAL, and Web of Science databases was conducted for comparative studies of pre- and post-vegetarian diet adoption combined with aerobic exercise interventions on glycemic control and body composition. Qualitative reviews and meta-analyses of fixed and random effects were conducted to pool available data. The results were validated by sensitivity analysis.

**Results:**

A total of 27 studies were selected for meta-analysis. Combining the studies included in the meta-analysis showed a mean difference for homeostasis model assessment of insulin resistance of − 0.75 (− 1.08 to − 0.42), fasting plasma glucose of − 0.27(− 0.30 to − 0.23), waist circumference of − 1.10 (− 5.06 to 2.86) and body mass index of − 0.70 (− 1.38 to − 0.01).

**Conclusion:**

In summary, our findings suggest that participants who adopted a vegetarian diet combined with aerobic exercise intervention had significantly lower fasting plasma glucose and insulin levels and improved body composition compared to preintervention participants.

**Level of evidence:**

Level I, systematic review and meta-analysis.

**Supplementary Information:**

The online version contains supplementary material available at 10.1007/s40519-023-01536-5.

## Introduction

Lifestyle and health-related behaviors, mainly including diet and physical activity, are strongly associated with global morbidity and mortality for many chronic diseases [1, 2]. Poor diet and lack of exercise are common high-risk lifestyle behaviors among people of all ages worldwide [3], which have led to increased rates of obesity, a major chronic disease that affects the overall health of humans. As the increase in the number of people with body mass index (BMI) in the overweight and obese range is closely related to the occurrence and development of chronic diseases, such as diabetes, metabolic syndrome, cardiovascular disease (CVD) and cancer, the prevention and treatment of obesity has become a global issue [4]. Growing evidence supports the role of diet, exercise, stress management, and smoking in the pathogenesis of metabolic diseases [5–7].

Lifestyle changes can alter the state of the body and prevent chronic diseases such as hypertension, diabetes, and CVD. What’s more, with more people being aware of the importance of environmental issues and compassion for animals and the potential health benefits of a vegetarian diet (VD), there has been a clear trend towards vegetarianism over the past few years [8]. VD [9, 10] is defined as a diet that excludes all animal products, including dairy products, eggs and honey. VD includes lacto-ovo-vegetarian diet (LOV), plant-based diet (PBD) and vegan diet, as well as some other dietary patterns. Compared to omnivorous diets, VD is rich in many vitamins, phytochemicals, and antioxidants, but lower in cholesterol, total fat, saturated fatty acids and serum vitamin B12 concentrations, especially omega-3 polyunsaturated fatty acids. This property is associated with lower blood pressure and BMI [11]. The impact of physical activity on human health and wellbeing has been demonstrated and is associated with a reduced risk of death [12]. Exercise recommendations include participation in regular moderate-intensity aerobic exercise for at least 150 min per week or more than 300 min per week to achieve long-term weight control and moderate-intensity weight lifting twice per week with 10 to 15 repetitions of resistance exercise [13].

The Pritikin Institute has conducted studies [14, 15] demonstrating improved glycemic control with a PBD combined with exercise as part of type 2 diabetes treatment. David and colleagues [15] demonstrated the potential benefits of a VD for glycemic control and insulin resistance (IR). Several meta-analyses [16–19] also confirmed the role of exercise in IR, glycemic control, and body composition. To our knowledge, there is no systematic review and meta-analysis on the effects of combined VD and aerobic exercise interventions on glycemic control, IR, and body composition. Therefore, we conducted a systematic literature search to summarize and synthesize the available data and performed a meta-analysis of published clinical trials to determine whether the combination of VD and exercise has additional benefits on glycemic control, IR, and body composition compared with VD or aerobic exercise alone.

## Materials

### Protocol and registration

This study is based on the PRISMA Statement Guidelines [20]. The protocol reviewed was preregistered with PROSPERO: CRD42022318778.

### Literature search strategy

In this meta-analysis, we considered only studies published in English. These studies were obtained by searching five electronic databases, namely: EBSCO, PubMed, Embase, Cochrane Central Register of Controlled Trials (CENTRAL), and Web of Science. The search was finished on September 4, 2022. The search strategy to identify all studies examining vegetarian diet combined with exercise interventions was (“plant-based diet”, “vegan diet”, “lacto-ovo-vegetarian diet” or “vegetarian diet”, etc.) and (“aerobic exercise”, “exercise”, or “exercise performance”). We searched “All field” in PubMed and for “subject terms” + “entry words”, “subject terms” in EBSCO, Embase, and Web of Science, and searched unrestrictedly in CENTRAL. In addition, we checked the reference lists of all primary studies and any relevant review articles for additional references. The search strategy of five databases is shown in Table S1 (Supplementary material 1 Table 1).

**Table 1:** Characteristics of studies included in the meta-analysis

Study	Country	Type of study	Participants characteristic/age/*N* (male/female)	Exercise intervention	Type of diet	Intervention duration
Ornish et al. [32]	The United States	RCT	coronary atherosclerosis patients /aged 56.1 ± 7.5 years/22(21/1)	Moderate aerobic exercise (typically walking)	low-fat VD	1 year
Null et al. [50]	The United States	Cohort study	People free of clinically overt disease/aged 31 to 78 years/52(23/29)	regular aerobic exercise (power-walking or jogging) 5d/wk, 75%HRmax, 30-45 min each time	a low-fat VD high in vitamins A, C, and E	6 months
Diehl et al. [45]	The United States	Cohort study	People were graduated and attended at least 80% of the educational lectures/aged 55 ± 11 years/ 288(123/165)	enhance daily exercise, walking or exercising 30 min a day	plantfood–centered diet	12 months
Ornish et al. [31]	The United States	RCT	patients with moderate to severe CHD/aged 57.4 ± 6.4 years /20(20/0)	aerobic exercise	10% fat whole foods VD	1 year
Toobert et al. [33]	The United States	RCT	Postmenopansal female and having documented CHD defined as atherosclerosis, myocardial infarction, percutaneous transluminal coronary angioplasty, and/or coronary bypass graft surgery /aged 64 ± 10 years/28 (0/28)	1 h session/day at least 3 days/wk week	very low-fat VD	24 months
Koertge et al. [48]	The United States	Cohort study	Patients with CAD/ aged 58.21 ± 10 years/440 (347/93)	moderate exercise (for 3 h/wk	low-fat, whole foods, PBD	1 year
Pischke et al. [35]	The United States	Non-RCT	People with CAD and DM (predominantly type 2) with those without DM/aged 58.39 ± 10.74 years/434(341/93)	1-h aerobic exercise session (e.g., on treadmills)	low-fat PBD	1 year
Pischke et al. [49]	The United States	Cohort study	CHD patients at risk for heart failure /aged 59.0 ± 9.8 years /181(148/33)	1 h aerobic exercise session (for example on a treadmill)	low-fat, PBD	12 months
Slavíček et al. [39]	Czech Republic	Non-RCT	Volunteers/ mean age 51 ± 14.5 years/1349(320/1029)	light physical training	low-fat, low-energy LOV	7 weeks
Marshall et al. [47]	The United States	Cohort study	military healthcare beneficiaries with CAD or CVD risk factors / aged 60.6 ± 9.7 years/142(101/41)	Aerobic exercise (180 min/wk)	LOV	1 year
Dod et al. [36]	The United States	Non-RCT	Participants with CAD or risk factors for CAD /mean age 56 years/27(14/13)	moderate exercise (3 h/wk)	PBD (10% calories from fat)	3 months
Telles et al. [52]	India	Cohort study	participants who had a BMI more than 30 kg/m^2^ /aged 40.3 ± 10.2 years /47(16/31)	Yoga: 5 h/day	a low fat, high fiber, VD	6 days
Chainani-Wu et al. [40]	The United States	Cohort study	the patients were diagnosed with CHD or with type 1 or T2D or were at high risk of CHD/aged 57.42 ± 8.6 years/125(51/74)	Engage in at least 3 h of aerobic exercise/wk and strength training activities at least 2 times/wk	low-fat, whole-foods, PBD	3 months
Kahleova et al. [27]	Czech Republic	RCT	subjects with T2D /age 30–70 years/BMI between 25 and 53 kg/m^2^/37(17/20)	No change in exercise habits within 12 wk; Exercise prescription was formulated from 13 to 24wk, 60%HRmax, 1 h/ time, 2 times /wk	VD	24 weeks
Kahleova et al. [28]	Czech Republic	RCT	subjects with T2D/age 30–70 years/BMI between 25 and 53 kg/m^2^/37(17/20)	No change in exercise habits within 12 wk; Exercise prescription was formulated from 13 to 24wk, 60%HRmax, 1 h/ time, 2 times /wk	VD	24 weeks
Kent et al. [41]	Australia	Cohort study	Individuals /mean age = 57.3 ± 12.9 years/ 5046(1690/3356)	engage in 30 min of daily moderate intensity physical activity	low-fat, PBD	30 days
Morton et al. [43]	The United States	Cohort study	Participants were representative of an at-risk population, with a mean BMI in the “obese” category, “prediabetic” fasting blood sugar levels, and elevated systolic blood pressure and low-density lipoprotein cholesterol levels/ aged 56.3 ± 12.1 years/971(311/660)	Daily exercise (30 min at moderate intensity or 10,000 steps)	whole grains, legumes, and fresh fruits and vegetables	30 days
Bhardwaj et al. [26]	India	RCT	obese vegetarian individuals /BMI > 25 kg/m^2^ to < 40 kg/m^2^/aged 38.9 ± 8.7 years/52(21/31)	exercise regime, on at least 6 out of the 7 days in a wk	VD	2 months
Lee et al. [30]	Korea	RCT	Young healthy adults/aged 20.0 ± 1.14 years /30(0/30)	aerobic, flexibility, and strength exercises (3 h/day)	VD	10 days
Kent et al. [42]	Australia	Cohort study	Individuals/aged 55.4 ± 16.3 years/22(10/22)	physical activity (advocating at least 30 min or 10,000 steps)	whole food, PBD	30 days
Cairo et al. [37]	The United States	Non-RCT	Women with curative-intent (stage 0–III) breast cancer who were not currently enrolled in any other wellness studies/aged 51.4 ± 8.1 years /127(0/127)	physical activity	PBD	12 months
Tsaban et al. [34]	Israel	RCT	presence of abdominal obesity or dyslipidaemia /aged 50.5 ± 10.8 years /98(87/11)	Physical activity	green Mediterranean diet (avoid red/processed meat consumption)	18 months
Ahrens et al. [46]	The United States	Cohort study	Adults/aged 46.89 + 12.38 years/73 (37 /36)	take part in daily exercise and yoga classes	PBD (with minimal sugar, salt, and oil)	6 days
Klimis et al. [29]	Australia	RCT	Adults are eligible if referred to a rapid access cardiology clinic for chest pain assessment, have a moderate-high absolute CVD risk (10-year Australian absolute CVD risk) ≥ 10%) / age:58.5 ± 10.4 years /124(53/71)	physical ability (full range of exercises; non-weight bearing exercises only; or unable to exercise)	VD	6 months
Suazo et al. [44]	Mexico	Cohort study	Most of them are overweight/aged 39.3 ± 15.9 years/13(8/5)	Morning exercise: 30 min walking + stretching; Walk 10 min after each meal; Aerobic + resistance 60 min per day	PBD	21 days
Świątkiewicz et al. [51]	Poland	Cohort study	Adults who underwent cardiac rehabilitation /aged 66.0 ± 9.0 years /101(69/32)	Structured and supervised exercise included regular, moderate aerobic and resistance/strength training	A whole food, low-fat, low in refined carbohydrates, nutritionally adequate, PBD (consisting of fruits, vegetables, whole grains, legumes, and soy products) without caloric restriction	9 weeks
Koeder et al. [38]	Germany	Non-RCT	Participants who physical and mental ability to take part in the study (self-reported) / aged 59.3 ± 0.9 years /93 (29/64)	physically active for at least 30 min per day,	Healthy PBD	1 year

*RCT* randomized controlled trial, *Non-RCT* non-randomized controlled trial, *BMI* body mass index, *HRmax* maximal heart rate, *CVD* cardiovascular disease, *CAD* coronary artery disease, *CHD* coronary heart disease, *DM* diabetes mellitus, *T2D* type 2 diabetes mellitus

Unit: min: minute; h: hour; wk: week

Diet types: VD: Vegetarian diet; LOV: lacto-ovo-vegetarian diet; PBD: Plant-based diet; UD: usual diet

### Study selection and eligibility criteria

#### Inclusion criteria

Studies were included if they met the following criteria: (1) Randomized controlled trials (RCTs), nonrandomized controlled trials (Non-RCTs), and cohort studies; (2) Written in English and translation of non-English articles into English; (3) The intervention was a combination of both vegetarian diet and exercise; (4) The outcome measures included one of the following: body composition, fasting plasma glucose (FPG), insulin, or homeostasis model assessment of insulin resistance(HOMA-IR); and (5) Availability of summary statistics for mean and standard deviation (SD), change in mean and SD, quartiles, or 95% confidence interval (CI) before and after the intervention for outcome measures.

#### Exclusion criteria

Studies were excluded if they met the following criteria: (1) Reviews, animal studies, comments, letters, books, conferences published in abstract form only; (2) No comparison based on pre- and postintervention. (3) Articles published in languages other than English. (4) Studies published later than the time of the literature search.

### Data extraction

The three authors (YL, JM-Y, HY) independently extracted data from the selected studies into Microsoft Excel spreadsheets; any disagreements were resolved at a consensus meeting. Table 1 presents a summary of the extracted data, which provides information on (1) Authors, year, and country of publication; (2) Study design; (3) Characteristics of the study population (i.e., physical health status, age, sample size, number of men and women); (4) Type of vegetarian intervention, and type of exercise intervention; and (5) Duration of intervention. One author (YL) extracted the data, and two authors (JM-Y, HY) checked and verified the extracted data.

### Quality assessment

Revman 5.4 software and the Cochrane Risk of Bias Assessment Tool [21] (RBT) were used to produce the literature risk of bias and quality for selected randomized controlled trials (RCTs). The risk of bias for cohort studies was assessed using the Newcastle Ottawa Observational Study Scale [21] (NOS). Nonrandomized controlled trials (Non-RCTs) were assessed using the methodological index for nonrandomized studies (MINORS) [22]. The assessment was first performed independently by two authors (YL, JM-Y), and when there was a lack of consensus, it was resolved by discussion and agreement with the third review author (HY).

### Statistical analysis

Meta-analysis was performed using Stata SE 15.0. Effect sizes were estimated using weighted mean differences (WMDs), standardized mean differences (SMDs), and 95% CIs. We used forest plots and *I*^2^ to assess heterogeneity between studies, where 0–40%, 30–60%, 50–90%, and 75–100% indicated possible irrelevance, moderate heterogeneity, substantial heterogeneity, and greater heterogeneity [23]. The fixed-effects model was used to assess studies with less heterogeneity, while the random-effects model was used to assess studies with greater heterogeneity. If the heterogeneity of the meta-results was high (*p* < 0.1 or/and *I*^2^ > 50%), subgroup and sensitivity analyses were produced to elucidate the sources of heterogeneity and to examine whether the risk of bias would alter the combined results. When more than seven articles were included, publication bias in the meta-analysis was detected by making funnel plots, Egger’s regression test [24], and Begg’s rank correlation test [25].

### Results

#### Study selection

**Fig. 1:**
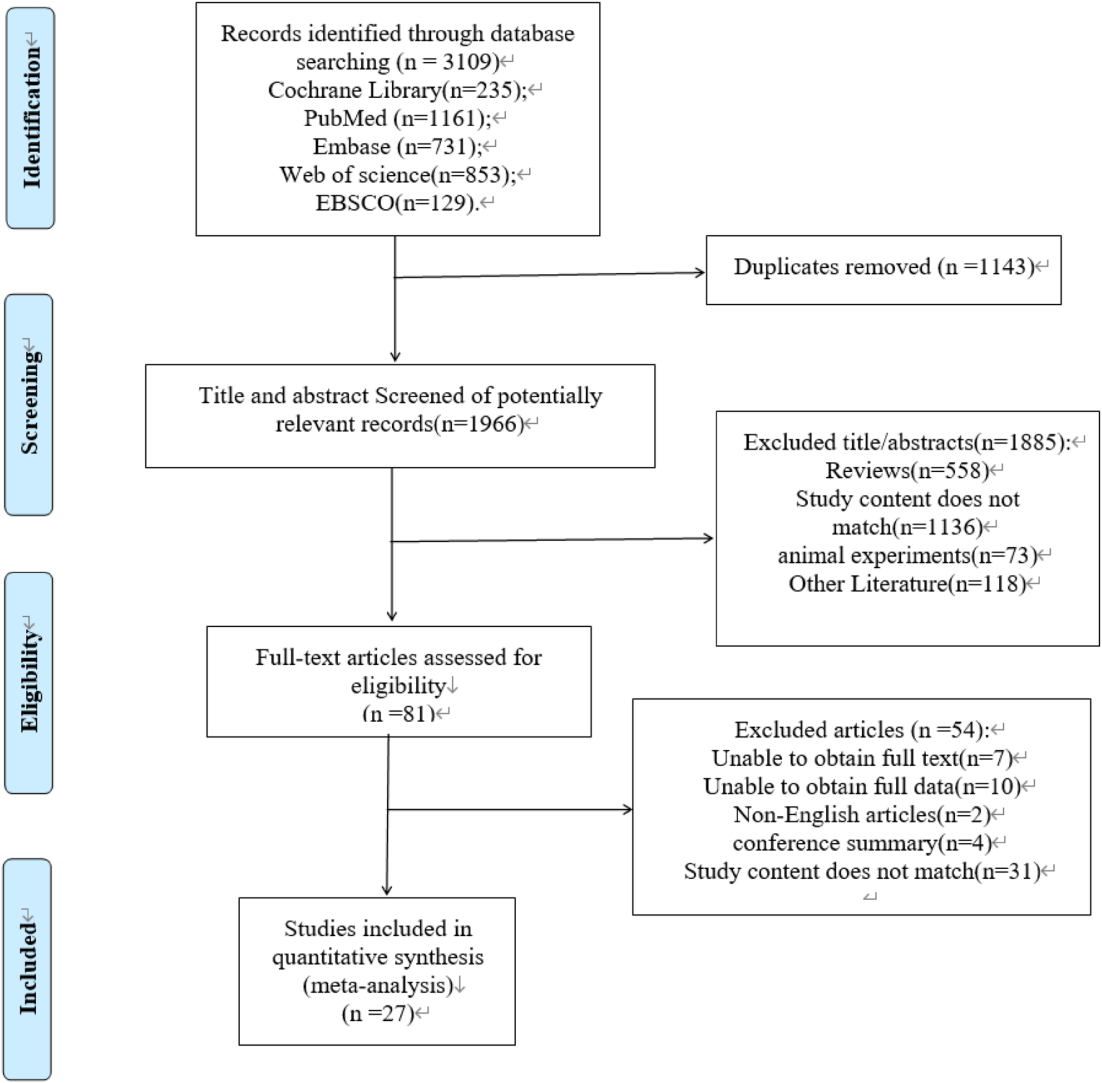
Flow chart search strategy

Data for eligible publications were searched until September 4, 2022. The literature search resulted in 3109 articles with 1161, 129, 731, 853, and 235 studies on PubMed, EBSCO, Embase, Web of Science, and the Cochrane Library, respectively. Exclusion of duplicates resulted in 1143 articles, of which the abstracts were screened for eligibility. A total of 1966 studies were retrieved from the full-text screening, and 27 studies were considered eligible for systematic evaluation and meta-analysis after screening by full-text reading. The process is shown in Fig. 1.

#### Excluded studies

Fifty-four studies were excluded after screening the full text. The most common reason was that the outcome indicator after the intervention was not body composition, FPG, insulin, or HOMA-IR; some articles were excluded because they did not fit the combined VD and aerobic exercise intervention, and others were excluded because they had more than just VD and exercise interventions but also added supplements, specific medications, etc. Seven articles were excluded because the full text was not available, and one article was excluded because it did not provide the data we needed and we were not able to rectify the situation even after contacting the original author numerous times.

#### Included studies

Details of the included study are shown in Table 1. The effect of a VD combined with an aerobic exercise intervention on people was systematically evaluated from the perspective of glucose metabolism and weight control by comparing postintervention measures with preintervention measures in the same subjects. Variables used as proxies for carbohydrate metabolism included FPG and IR; body weight (BW), BMI, waist circumference (WC), and hip circumference (HC) for body composition measures.

### Study characteristics

A total of 27 studies with a total of 9053 subjects were included in this paper, and in general, more female subjects than male subjects were included. Fourteen interventional studies and thirteen observational studies were included. The intervention studies were subdivided into 9 RCTs and 5 non-RCTs, and the observational studies included cohort studies. The sample sizes ranged from 12 to 5046 individuals, and the study durations ranged from 6 days to 24 months.

Three studies included only female subjects, three studies included only male subjects, 13 studies included healthy subjects, and 14 studies included patients with metabolic abnormalities such as overweight or obese BMI, coronary artery disease (CAD), diabetes, or hypertension. All studies were published between 1990 and 2022, and approximately two-thirds of the included studies were published after 2010. All of them were published in India, Mexico, Germany, Australia, Italy, Korea, Canada, Israel, the Czech Republic, Poland and the United States, with nearly half of the studies published in the United States.

#### Results from quality assessments

**Fig. 2:**
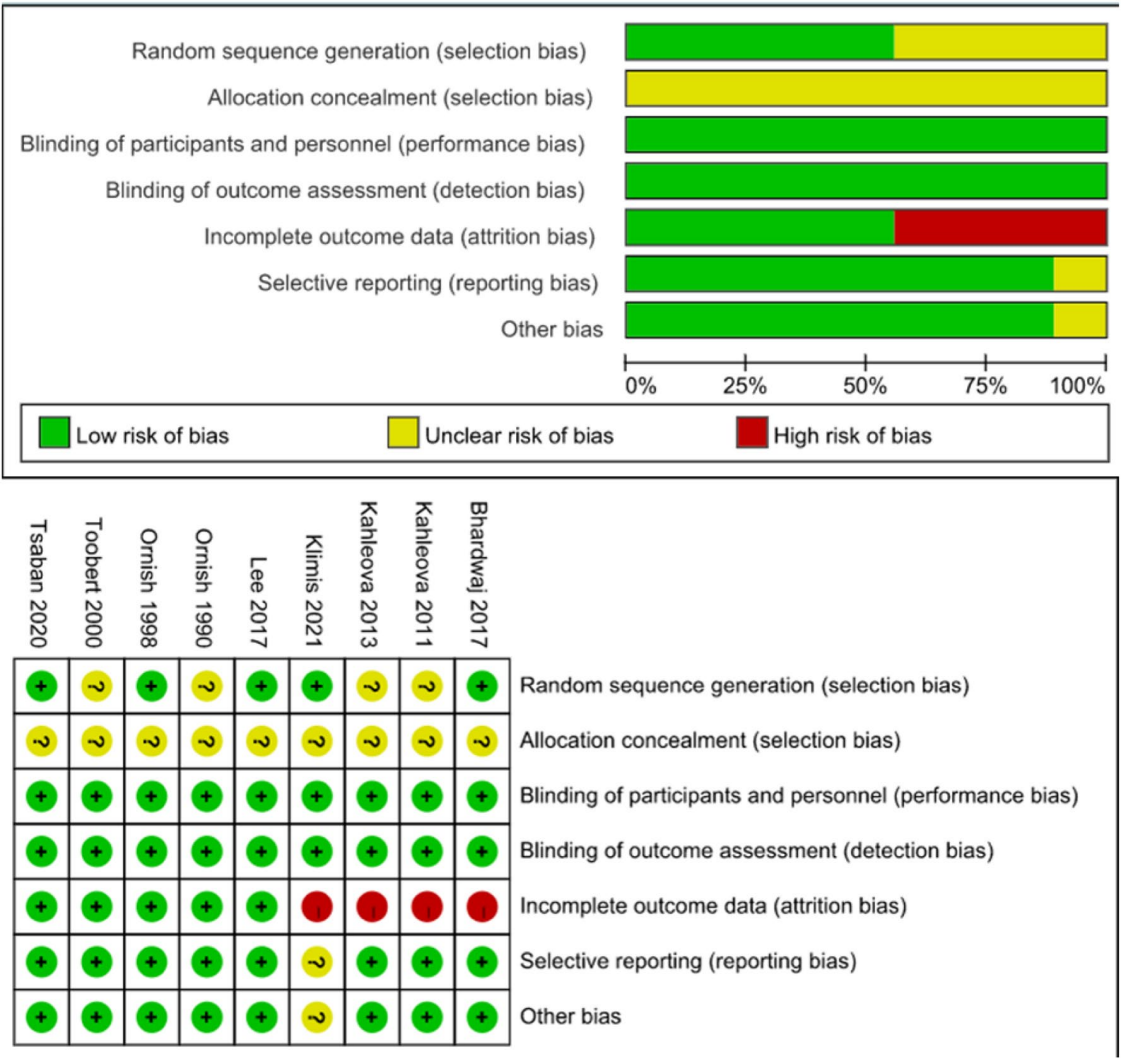
Represents the Risk of bias (ROB) results of the graph and summary for included RCTs. In the picture above, green represents low risk, yellow represents unclear risk, red represents a high risk

The risk of bias graph in RevMan lists the risk of bias for the nine RCTs [26–34]. The results indicate that the included RCTs were of high quality. The details are shown in Fig. 2. The risk of bias for the five included non-RCT studies [35–39] is presented in Table S2. According to the MINORS entry, one studies [37] showed a high risk of bias, with overall scores of 11, because many details of the study protocol were not reported in the publication. The other four studies had an overall low to moderate risk of bias, with total scores of 14, 15, 16, and 16, respectively. Points were deducted from total scores due to a lack of prospective data collection details, statistical analysis methods, recruitment of participants, blinding of results, and problems with using participants as their own controls and parameters being estimated at two different time points. The quality of the literature was assessed for the thirteen cohort studies, indicating that the quality of the literature was high quality and the overall risk was low, details of which can be found in Table S3.

#### Effect of intervention on glycemic control

**Fig. 3:**
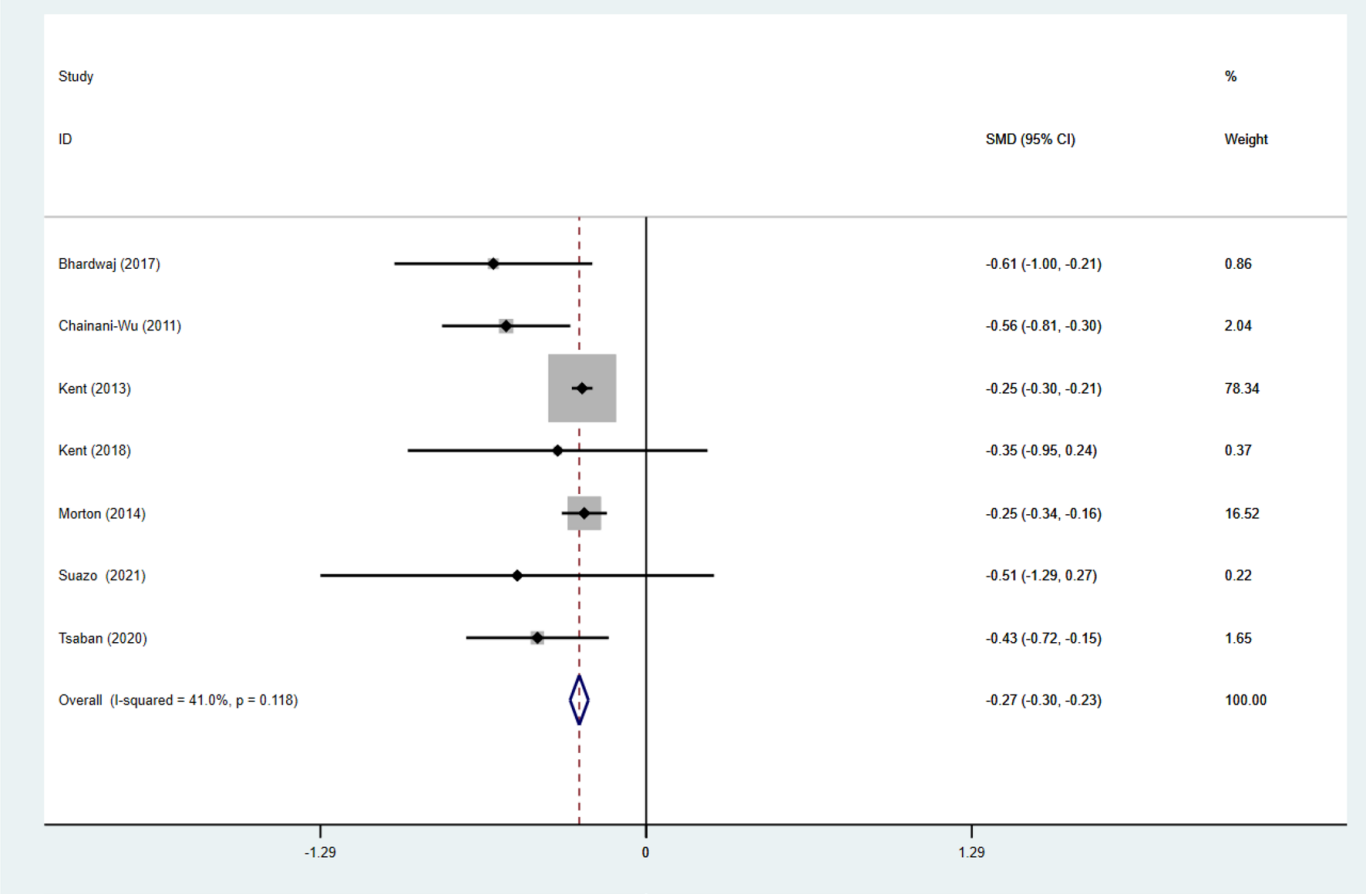
Forest figure of FPG. For each study, squares represent the mean difference in intervention effects, with horizontal lines intersecting them as the lower and upper limits of the 95% CI. The size of each square represents the relative weight of the studies conducted in the meta-analysis. The diamond represents the results of the meta-analysis combining the individual studies

**Fig. 4:**
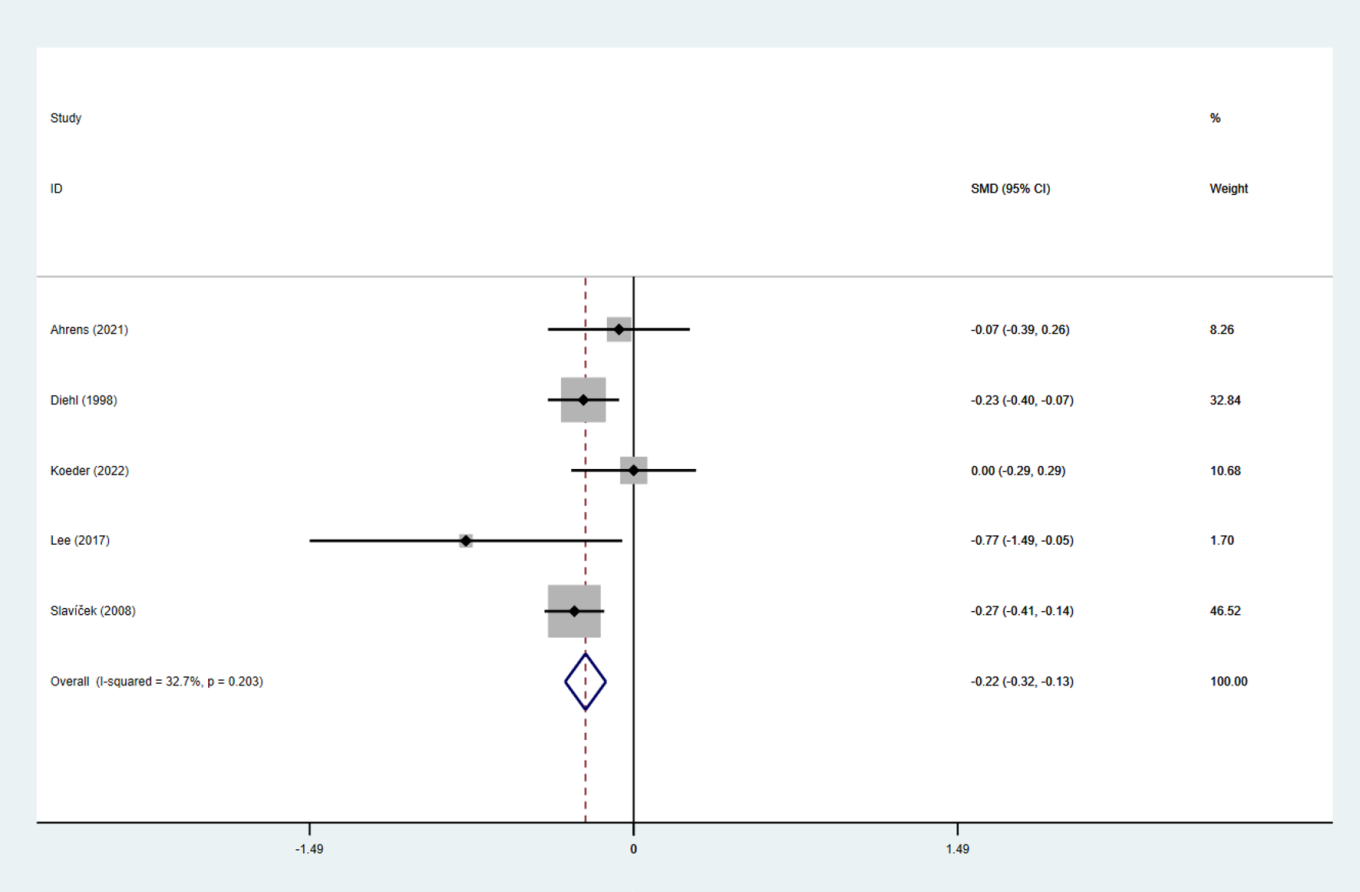
Forest figure of glucose. For each study, squares represent the mean difference in intervention effects, with horizontal lines intersecting them as the lower and upper limits of the 95% CI. The size of each square represents the relative weight of the studies conducted in the meta-analysis. The diamond represents the results of the meta-analysis combining the individual studies

Seven studies [26, 34, 40–44] reported FPG data for 5862 subjects, and five studies [30, 38, 39, 45, 46]reported glucose data for 878 subjects. Pooled results under a fixed effects model showed that individuals adhering to the VD and aerobic exercise interventions had significantly lower FPG (SMD: − 0.27; 95% CI: − 0.30 to − 0.23; *p* < 0.001; *I*^2^: 41%) and a random effects model showed nonsignificant changes in glucose (SMD: − 0.22; 95% CI − 0.32 to − 0.13; *p* < 0.001; *I*^2^: 32.7%). There was significant heterogeneity in both groups (Figs. 3 and 4).

### Effect of intervention on IR

**Fig. 5:**
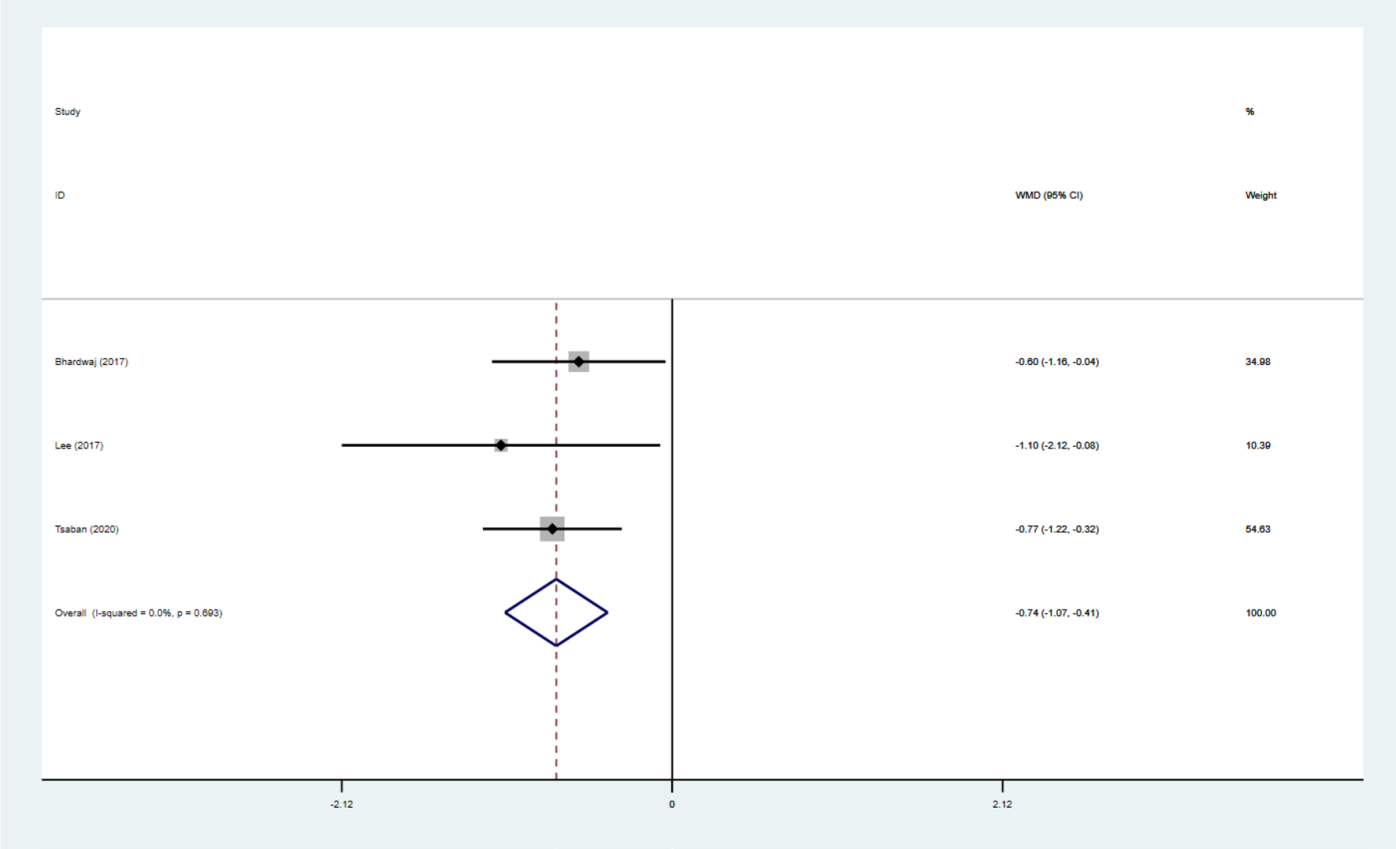
Forest Figure of HOMA-IR. For each study, squares represent the mean difference in intervention effects, with horizontal lines intersecting them as the lower and upper limits of the 95% CI. The size of each square represents the relative weight of the studies conducted in the meta-analysis. The diamond represents the results of the meta-analysis combining the individual studies

**Fig. 6:**
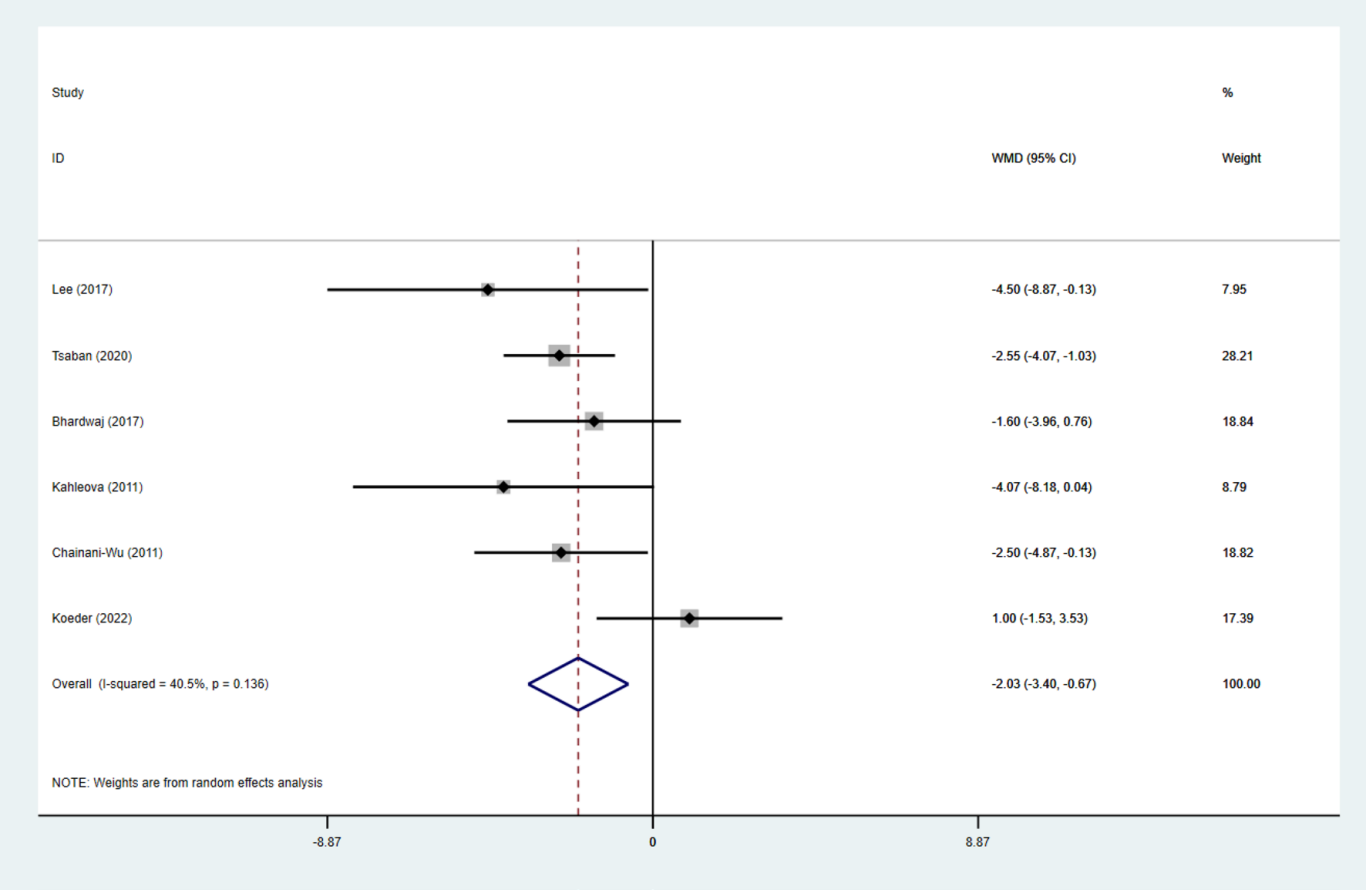
Forest Figure of insulin. For each study, squares represent the mean difference in intervention effects, with horizontal lines intersecting them as the lower and upper limits of the 95% CI. The size of each square represents the relative weight of the studies conducted in the meta-analysis. The diamond represents the results of the meta-analysis combining the individual studies

Three studies [26, 30, 34] reported HOMA-IR data for 166 subjects, and six studies [26, 27, 30, 34, 38, 40] reported insulin data for 419 subjects. The pooled results under a fixed-effects model showed that HOMA-IR scores were significantly lower in individuals who adhered to a VD combined with exercise intervention (WMD: − 0.75; 95% CI − 1.08 to − 0.42; *p* < 0.001; *I*^2^:0%). Insulin data for study individuals were then used in a random-effects model, and the pooled results showed significantly lower insulin levels (WMD: − 2.03; 95% CI − 3.40 to − 0.67; *p* = 0.003; *I*^2^:40.5%) (Figs. 5 and 6).

#### Effect of intervention on body composition

Twenty studies [26–28, 30–33, 35–39, 44–51] reported data on BW in 3152 subjects. Eighteen studies [26, 28–30, 33, 37–47, 51, 52] reported data on BMI in 7574 subjects. Eight studies [26, 27, 29, 34, 38, 46, 51, 52] reported data on waist circumference (WC) in 624 subjects. Four studies [26, 46, 52, 53] reported data on the hip circumference (HC) in 177 subjects. Six studies [26, 30, 35, 47, 49, 51] reported data on body fat percentage (BF%) in 923 subjects.

Pooled results under a fixed-effects model indicated that individuals adhering to a VD combined with exercise intervention had a nonsignificant change in HC was significantly lower (WMD: − 2.55; 95% CI − 4.04 to − 1.05; *p* = 0.001; *I*^2^: 0%). No heterogeneity was found in HC of the integration of results under the fixed effects model.

**Fig. 7:**
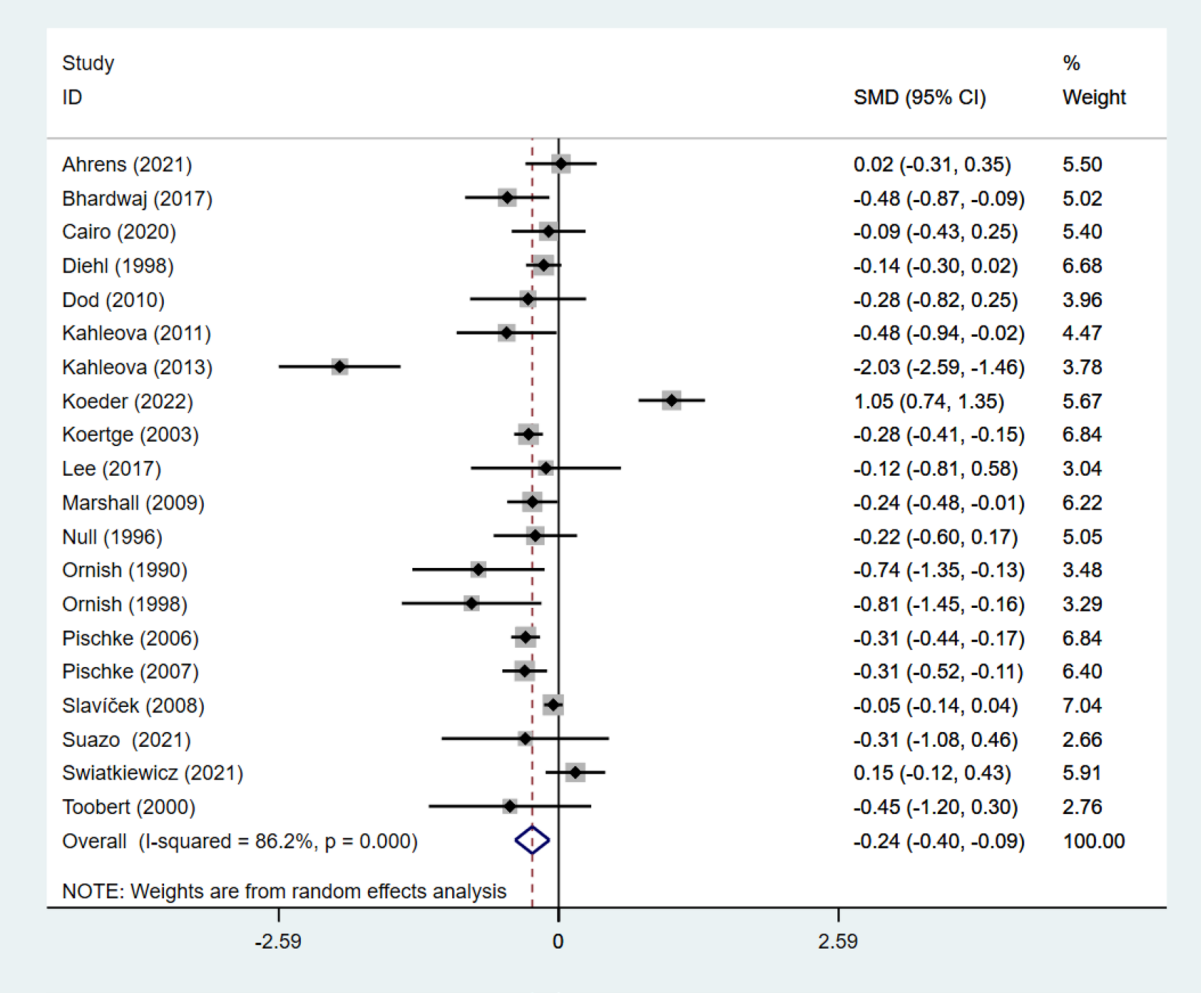
Forest Figure of BW. For each study, squares represent the mean difference in intervention effects, with horizontal lines intersecting them as the lower and upper limits of the 95% CI. The size of each square represents the relative weight of the studies conducted in the meta-analysis. The diamond represents the results of the meta-analysis combining the individual studies

**Fig. 8:**
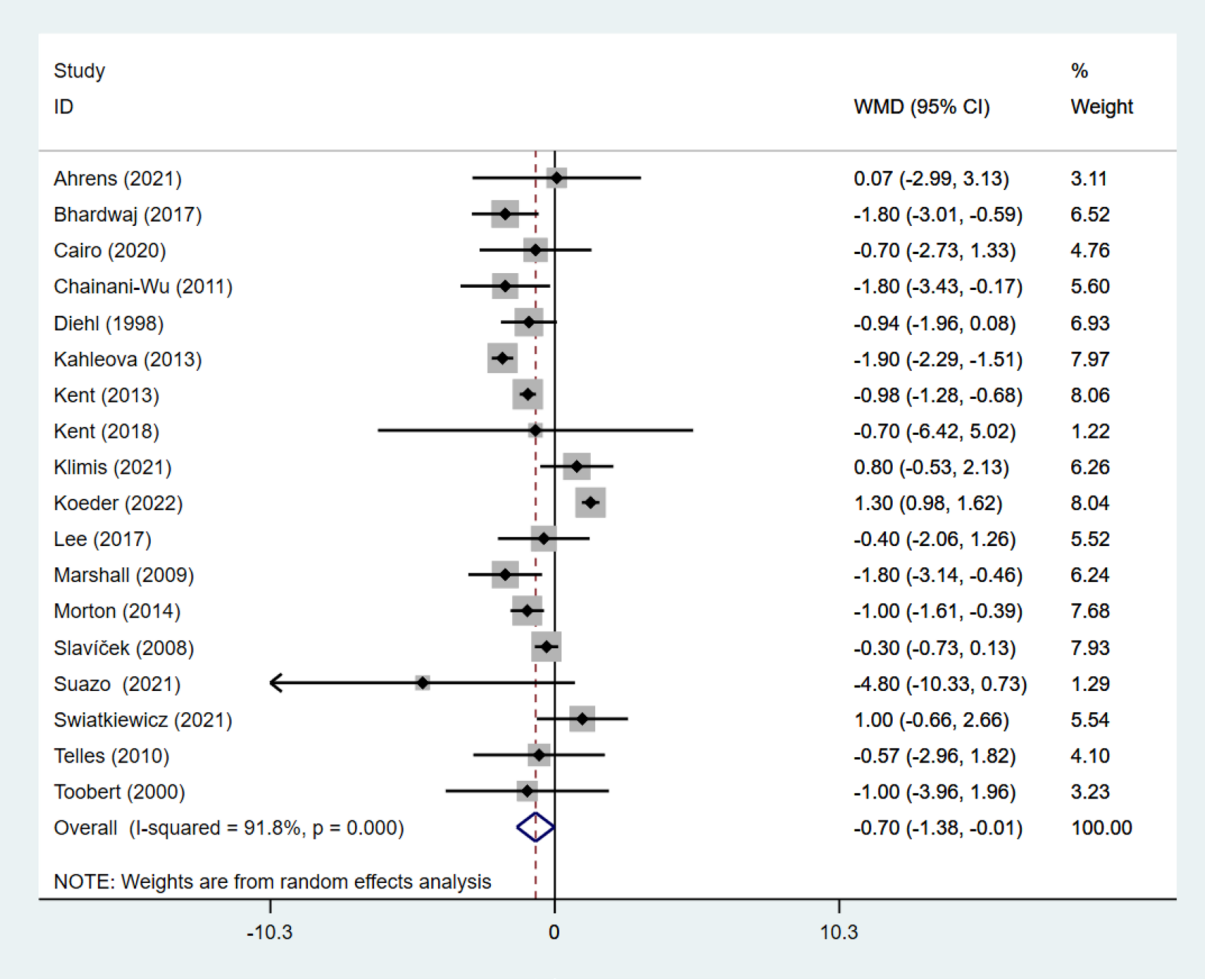
Forest Figure of BMI. For each study, squares represent the mean difference in intervention effects, with horizontal lines intersecting them as the lower and upper limits of the 95% CI. The size of each square represents the relative weight of the studies conducted in the meta-analysis. The diamond represents the results of the meta-analysis combining the individual studies

**Fig. 9:**
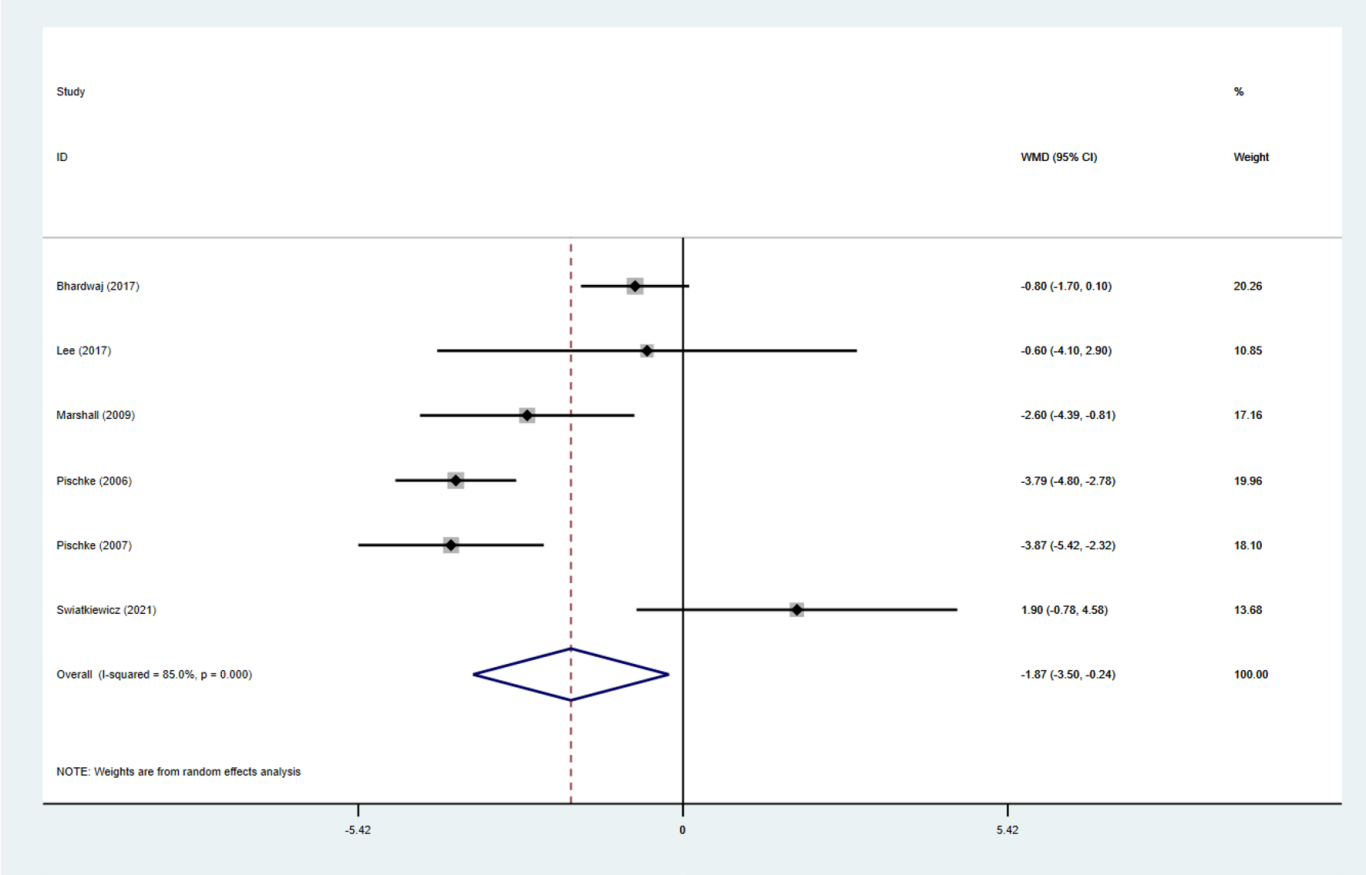
Forest Figure of BF%. For each study, squares represent the mean difference in intervention effects, with horizontal lines intersecting them as the lower and upper limits of the 95% CI. The size of each square represents the relative weight of the studies conducted in the meta-analysis. The diamond represents the results of the meta-analysis combining the individual studies

**Fig. 10:**
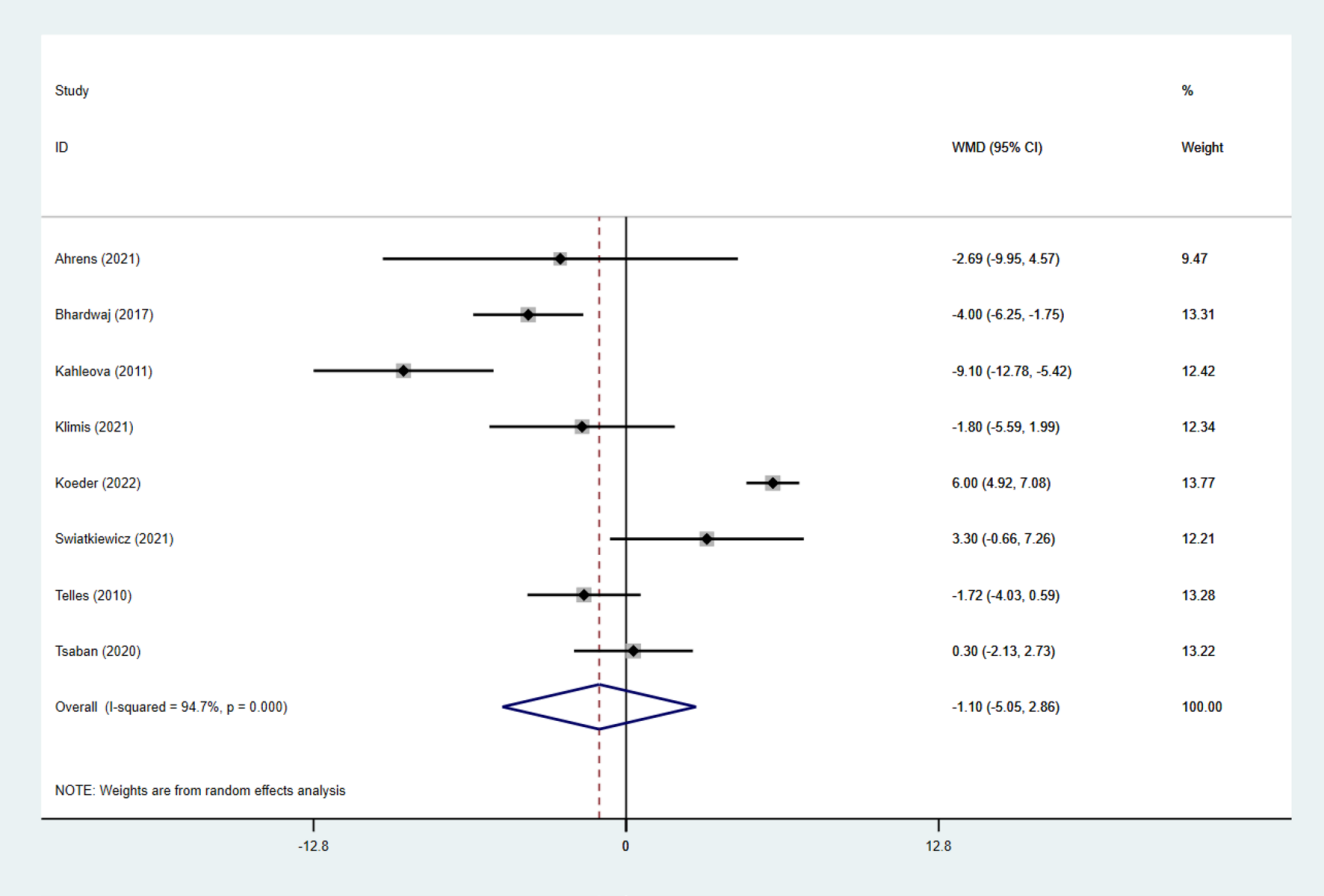
Forest Figure of WC. For each study, squares represent the mean difference in intervention effects, with horizontal lines intersecting them as the lower and upper limits of the 95% CI. The size of each square represents the relative weight of the studies conducted in the meta-analysis. The diamond represents the results of the meta-analysis

For the study individuals, BW, BMI, BF% and WC were used in a random effects model, and the combined results showed a significant reduction in BW (SMD: − 0.24; 95% CI − 0.40 to − 0.09; *p* = 0.003; *I*^2^: 86.2%), a significant reduction in BMI (WMD: − 0.70; 95% CI − 1.38 to − 0.01; *p* = 0.046; *I*^2^:91.8%). BF% was significantly reduced (WMD: − 1.87; 95% CI − 3.50 to − 0.24; *p* = 0.025; *I*^2^: 85.0%), and WC was also significantly reduced (WMD: − 1.10; 95% CI − 5.06 to 2.86; *p* = 0.02; *I*^2^:94.7%). Substantial heterogeneity was present in all groups (Figs. 7, 8, 9 and 10).

To elucidate substantial heterogeneity in body composition, subgroup analyses were [31, 36]. Heterogeneity increased in the ≥ 1-month subgroup and decreased in the < 1-month subgroup, suggesting that the duration of intervention may be a source of heterogeneity. (Supplementary material 2: Figs. 1, 2, 3, and 4).

### Sensitivity analysis

The results of the sensitivity analysis were stable, with no significant effect of any individual effect size on the overall effect size of all parameters (Supplementary material 2: Figs. 5, 6, and 7).

### Publication bias

Begg’s test demonstrated no publication bias for FPG (*p* = 0.548), glucose (*p* = 1.000), insulin (*p* = 0.452), HOMA-IR (*p* = 1.000), BW (*p* = 0.347), BMI (*p* = 0.363), HC (*p* = 0.734), WC (*p* = 0.711), or BF% (*p* = 0.707). Egger’s test showed no significant publication bias for all indicators except weight. Visual inspection of the funnel plot yielded the same results (Supplementary material 2: Figs. 8, 9, and 10).

### Discussion

#### Effect of the vegetarian diet combined with aerobic exercise

The results of our systematic review and meta-analysis showed that VD combined with aerobic exercise intervention had variable effects on glycemic control and IR in various populations compared to preintervention, mainly in terms of significant reductions in FPG, HOMA-IR and insulin levels, and nonsignificant reductions in glucose values. The effects of a VD on glycemic management, IR, and body composition have been confirmed by several clinical trials and narrative reviews. The addition of aerobic exercise training has been shown to further enhance the improving effects of a VD, it can reduce diabetes and its complications caused by chronic glycotoxicity caused by persistent hyperglycemia [27, 54].

The combination of VD and exercise interventions reduced body composition levels, including laboratory measures (weight, body fat, BF%) and anthropometric measures (HC and WC), and the reduction in anthropometric measures was generally greater than that in laboratory measures. In subgroup analyses, the effects of intervention duration ≥ 1 month and < 1 month were statistically significant, and the effects were more pronounced for intervention duration < 1 month. We observed a significant reduction in WC and HC in all subjects compared to the preintervention baseline values. This was mainly associated with a reduction in the level of adipose tissue, which confirms the effect of vegetarianism and exercise [55], with an improvement in metabolic profile consistent with a reduction in body weight, fat mass, and WC. Previous studies have shown that lower upper body fat positioning is associated with a lower incidence of glucose tolerance and IR [56, 57]. The reduction in WC in the absence of changes in weight and body composition is clinically meaningful because WC is inversely associated with the risk of chronic disease [58, 59].

A study [30] showed that in healthy subjects, BW, BMI, FPG, and HOMA-IR levels were significantly lower with a VD combined with exercise intervention compared to a control group on a regular diet and no exercise. In two RCTs of obese individuals, WC, FPG, and HOMA-IR levels were significantly lower in the VD group with the same level of exercise, and the decrease in WC was greater than that in the control group [34]. The results of another RCT [26] showed a greater decrease in FPG levels after the intervention than in the control group. In another study [60] investigating the effects of diet and exercise (alone or in combination) on weight and body composition in obese menopausal women, there was a significant weight loss effect in the diet and moderate-intensity aerobic exercise groups, but the greatest change was observed in the group where the two interventions were used in combination. Together, these studies demonstrate that the combined effect of a VD and exercise is more effective than exercise alone.

Lee [30] suggested that short-term lifestyle programs, including diet and exercise, can improve the physical health and physical fitness of healthy adults, which could explain the heterogeneity that can be delineated by the duration of the intervention. Heterogeneity decreased in all subgroup analyses where the duration of intervention was less than one month. Interestingly, in the subgroup analysis, only the BF% increased and was greater than 50% of the heterogeneity for interventions < 1 month. The rest of the metrics had zero heterogeneity for intervention times < 1 month, while the heterogeneity was large for intervention times ≥ 1 month. In addition, some studies have suggested that vegetarians may not be able to exercise optimally at high intensity because they do not eat meat and therefore have lower creatine concentrations [61]. However, short-term vegetarian diets and aerobic exercise programs have been shown to improve skeletal muscle strength, insulin sensitivity, and blood lipids, as well as aid in weight loss and alter body composition. For example, a previous study reported that a 14-day diet and exercise intervention reduced BMI and insulin sensitivity [62].

The vast majority of the included studies indicated that subjects had lower BW and BMI after the intervention compared to preintervention, and the results were statistically significant. Only two studies [46, 63] demonstrated an increase rather than a decrease in BW after the intervention compared to the preintervention period. The only study of elevated BMI, with a six-day clinical trial led by Ahrens et al. [46], showed substantial improvements in lipid profiles and blood pressure associated with cardiovascular disease, although the changes in BW and BMI were minimal.

The beneficial effects of a VD with aerobic exercise training may be related to several possible mechanisms. Exercise training in individuals with an overweight or obese BMI, including aerobic and moderate intensity training, enhances mitochondrial function, and increases mitochondrial volume and protein turnover (due to impaired protein degradation and new functional protein synthesis), skeletal muscle changes in metabolic enzymes, the capillary to muscle fiber ratio, and insulin sensitivity. In turn, exercise training decreases catabolic mRNA expression, cardiac changes, and catheter artery changes, leading to improved cardiovascular health [64]. PBD may contain low levels of saturated fats (SFAs), mainly from oils such as palm oil and coconut oil [65]. The accumulation of free fatty acid (FFA) intermediates, ceramides, diacylglycerols, and SFAs, especially palmitates, can inhibit insulin signaling in myocytes at the cytosolic level [66]. In addition, excessive palmitate oxidation promotes mitochondrial dysfunction, which reduces ATP synthesis, thereby decreasing the bioavailability of ATP for insulin signaling and increasing oxidative stress [67, 68].

### Strengths and limitations

The current meta-analysis has several strengths and limitations. The strengths are that this meta-analysis included a total of 9053 participants, regardless of gender or race and physical health status, with broad coverage, making the study results more reliable.

Some limitations remain in our study. RCTs are considered the gold standard for determining causality findings; However, there are fewer RCTs of vegetarian diets combined with aerobic exercise interventions published in the literature based on our inclusion criteria. Nearly one-third of the included literature was cross-sectional studies. Thus, the study is less informative and does not allow for causal inferences and is susceptible to limitations common to all observational studies, including recommendation bias, self-reported adherence measures, and low screening-to-enrollment rates[47].

Another limitation of this systematic review and meta-analysis is that the studies provide different definitions of different types of VD but do not discuss and analyze each type of VD in separate groups but rather combine data from PBD, low-fat PBD, LOV, and VD for a unified analysis. Since individual studies did not report data separately for men and women, our study did not use gender as a classification criterion but rather combined them for discussion. Therefore, it is uncertain whether the effects of VD combined with exercise interventions on glycemic control, IR, and body composition were the same in men and women. Only a limited number of studies included in the current analysis focused on the effects of vegetarian diets and exercise interventions on insulin and HOMA-IR. In addition, with less than one-third of the studies included in this systematic review and meta-analysis being classified RCTs, the level of evidence-based data is greatly reduced.

### What is already known on this subject?

In recent years, a vegetarian and aerobic lifestyle has received increasing interest. Its benefits to the body are numerous, including glycemic control, body composition regulation and IR.

### What does this study add?

While the effects of a single VD or single aerobic exercise on body composition have been described, this is the first meta-analysis of the effects of a vegetarian diet combined with aerobic exercise on body composition improvement, which demonstrates that the combined effect of the two is greater than the effect of a large mono-vegetarian diet or single exercise.

### Conclusion

In conclusion, our study provides evidence that a VD combined with exercise intervention can be effective in glycemic control, IR, and improving body composition. We suggest that rather than focusing only on what to eat or how to exercise, a more comprehensive community-based lifestyle intervention program should be considered that focuses on promoting a VD and encouraging daily moderate-intensity physical activity. Just as multiple factors interact to affect the health of all individuals, comprehensive lifestyle interventions may improve the health status of the entire population. This has important public health implications for interventions to treat abnormal FPG and IR, to control obesity, and especially for the prevention and management of CVD and diabetes, among others.

## Supplementary Information

Below is the link to the electronic supplementary material.

Supplementary file1 (DOCX 29 KB)

Supplementary file2 (DOC 888 KB)

## Data Availability

Published data used for the systematic review and meta-analysis are available from the authors.
