# Supplementary material for: Effects of a vegetarian diet combined with aerobic exercise on glycemic control, insulin resistance, and body composition: a systematic review and meta-analysis
Source: Eat Weight Disord. 2023 Feb 15;28(1):9. doi: 10.1007/s40519-023-01536-5 (PMC9931794; doi:10.1007/s40519-023-01536-5)
Supplement: Supplementary file 1 — Supplementary file1 (DOCX 29 KB) [file 40519_2023_1536_MOESM1_ESM.docx]

**Supplementary Table1:** **Search strategies of PubMed、cochrane、web of science、 Embase、EBSCO.**

**Search strategies of** PubMed

#1 (Exercises) OR (Physical Activity) OR (Activities, Physical) OR (Activity, Physical) OR (Physical Activities) OR (Exercise, Physical) OR (Exercises, Physical) OR (Physical Exercise) OR (Physical Exercises) OR (Acute Exercise) OR (Acute Exercises) OR (Exercise, Acute) OR (Exercises, Acute) OR (Exercise, Isometric) OR (Exercises, Isometric) OR (Isometric Exercises) OR (Isometric Exercise) OR (Exercise, Aerobic) OR (Aerobic Exercise) OR (Aerobic Exercises) OR (Exercises, Aerobic) OR (Exercise Training) OR (Exercise Performance)

#2  (Diets, Vegetarian) OR (Vegetarian Diets) OR (Vegetarian Diet) OR (Lacto-Vegetarian Diet) OR (Diet, Lacto-Vegetarian) OR (Diets, Lacto-Vegetarian) OR (Lacto Vegetarian Diet) OR (Lacto-Vegetarian Diets) OR (Plant-Based Diet) OR (Diets, Plant-Based) OR (Plant Based Diet) OR (Plant-Based Diets) OR (Diet, Plant-Based) OR (Diet, Plant Based) OR (Plant-Based Nutrition) OR (Nutrition, Plant-Based) OR (Plant Based Nutrition) OR (Lacto-Ovo Vegetarian Diet) OR (Diet, Lacto-Ovo Vegetarian) OR (Diets, Lacto-Ovo Vegetarian) OR (Lacto Ovo Vegetarian Diet) OR (Lacto-Ovo Vegetarian Diets) OR (Vegetarian Diet, Lacto-Ovo) OR (Vegetarian Diets, Lacto-Ovo) OR (Vegetarianism) OR (Diets, Vegan) OR (Vegan Diets) OR (Vegan Diet) OR (Veganism)

#3 "Exercise"[Mesh]

#4 "Diet, Vegetarian"

#5 "Diet, Vegan"[Mesh]

#6 #1 OR #3

#7 #2 OR #4 OR #5

#8 #6 AND #7

#9 fliters：RCTs AND CTs

**Search strategies of** Cochrane

#1 MeSH descriptor: [Diet, Vegan] explode all trees

#2 MeSH descriptor: [Diet, Vegetarian] explode all trees

#3 MeSH descriptor: [Exercise] explode all trees

#4 (Diets, Vegetarian):ti,ab,kw OR (Vegetarian Diets):ti,ab,kw OR (Vegetarian Diet):ti,ab,kw OR ( Lacto-Vegetarian Diet):ti,ab,kw OR (Diet, Lacto-Vegetarian):ti,ab,kw OR (Diets, Lacto-Vegetarian):ti,ab,kw OR (Lacto Vegetarian Diet):ti,ab,kw OR ( Lacto-Vegetarian Diets):ti,ab,kw OR ( Plant-Based Diet):ti,ab,kw OR (Diets, Plant-Based):ti,ab,kw OR (Plant Based Diet):ti,ab,kw OR ( Plant-Based Diets):ti,ab,kw OR (Diet, Plant-Based):ti,ab,kw OR ( Diet, Plant Based):ti,ab,kw OR (Plant-Based Nutrition):ti,ab,kw OR (Nutrition, Plant-Based):ti,ab,kw OR (Plant Based Nutrition):ti,ab,kw OR ( Lacto-Ovo Vegetarian Diet):ti,ab,kw OR (Diet, Lacto-Ovo Vegetarian):ti,ab,kw OR (Diets, Lacto-Ovo Vegetarian):ti,ab,kw OR (Lacto Ovo Vegetarian Diet):ti,ab,kw OR (Lacto-Ovo Vegetarian Diets):ti,ab,kw OR (Vegetarian Diet, Lacto-Ovo):ti,ab,kw OR ( Vegetarian Diets, Lacto-Ovo):ti,ab,kw OR (Vegetarianism):ti,ab,kw OR (ovo-vegetarian):ti,ab,kw OR (Diets, Vegan):ti,ab,kw OR (Vegan Diets):ti,ab,kw OR (Vegan Diet):ti,ab,kw (Veganism)

#5 (Exercises):ti,ab,kw OR ( Physical Activity):ti,ab,kw OR (Activities, Physical):ti,ab,kw OR ( Activity, Physical):ti,ab,kw OR ( Physical Activities):ti,ab,kw OR ( Exercise, Physical):ti,ab,kw OR (Exercises, Physical):ti,ab,kw OR ( Physical Exercise):ti,ab,kw OR (Physical Exercises):ti,ab,kw OR (Acute Exercise):ti,ab,kw OR ( Acute Exercises):ti,ab,kw OR ( Exercise, Acute):ti,ab,kw OR (Exercises, Acute):ti,ab,kw OR ( Exercise, Isometric):ti,ab,kw OR ( Exercises, Isometric):ti,ab,kw OR ( Isometric Exercises):ti,ab,kw OR (Isometric Exercise):ti,ab,kw OR ( Exercise, Aerobic):ti,ab,kw OR ( Aerobic Exercise):ti,ab,kw OR ( Aerobic Exercises):ti,ab,kw OR (Exercises, Aerobic):ti,ab,kw OR (Exercise Training):ti,ab,kw OR ( Exercise Trainings):ti,ab,kw OR (Training, Exercise):ti,ab,kw OR (Trainings, Exercise):ti,ab,kw OR (Exercise Performance)

#6 #1 OR #2 OR #4

#7 #3 OR #5

#8 #6 AND #7

**#9 filter: clinical trials**

**Search strategies of** Web of Science

#1 TS=( (Diets, Vegetarian) OR (Vegetarian Diets) OR (Vegetarian Diet) OR (Lacto-Vegetarian Diet) OR (Diet, Lacto-Vegetarian) OR (Diets, Lacto-Vegetarian) OR (Lacto Vegetarian Diet) OR (Lacto-Vegetarian Diets) OR (Plant-Based Diet) OR (Diets, Plant-Based) OR (Plant Based Diet) OR (Plant-Based Diets) OR (Diet, Plant-Based) OR (Diet, Plant Based) OR (Plant-Based Nutrition) OR (Nutrition, Plant-Based) OR (Plant Based Nutrition) OR (Lacto-Ovo Vegetarian Diet) OR (Diet, Lacto-Ovo Vegetarian) OR (Diets, Lacto-Ovo Vegetarian) OR (Lacto Ovo Vegetarian Diet) OR (Lacto-Ovo Vegetarian Diets) OR (Vegetarian Diet, Lacto-Ovo) OR (Vegetarian Diets, Lacto-Ovo) OR (Vegetarianism) OR (Diets, Vegan) OR (Vegan Diets) OR (Vegan Diet) OR (Veganism) OR (Diet, Vegetarian) OR (Diet, Vegan))

**#2 TS=((Exercise) OR (Exercises) OR (Physical Activity) OR (Activities, Physical) OR (Activity, Physical) OR (Physical Activities) OR (Exercise, Physical) OR (Exercises, Physical) OR (Physical Exercise) OR (Physical Exercises) OR (Acute Exercise) OR (Acute Exercises) OR (Exercise, Acute) OR (Exercises, Acute) OR (Exercise, Isometric) OR (Exercises, Isometric) OR (Isometric Exercises) OR (Isometric Exercise) OR (Exercise, Aerobic) OR (Aerobic Exercise) OR (Aerobic Exercises) OR (Exercises, Aerobic) OR (ExerciseTraining) OR (Exercise Performance))**

**#3 #1 AND #2**

#4 TS=((Randomized Controlled Trial OR Randomized Controlled Trials OR RCT OR RCTs OR CCT OR CCTs OR Clinical trials OR Clinical trial))

#5 #3 AND #4

**Search strategies of** Embase

#1'exercise'/exp

#2 'vegetarian diet'/exp

#3 'vegan diet'/exp

#4 'exercises':ab,ti OR 'physical activity':ab,ti OR 'activities, physical':ab,ti OR 'activity, physical':ab,ti OR 'physical activities':ab,ti OR 'exercise, physical':ab,ti OR 'exercises, physical':ab,ti OR 'physical exercise':ab,ti OR 'physical exercises':ab,ti OR 'acute exercise':ab,ti OR 'acute exercises':ab,ti OR 'exercise, acute':ab,ti OR 'exercises, acute':ab,ti OR 'exercise, isometric':ab,ti OR 'exercises, isometric':ab,ti OR 'isometric exercises':ab,ti OR 'isometric exercise':ab,ti OR 'exercise, aerobic':ab,ti OR 'aerobic exercise':ab,ti OR 'aerobic exercises':ab,ti OR 'exercises, aerobic':ab,ti OR 'exercise training':ab,ti OR 'exercise trainings':ab,ti OR 'training, exercise':ab,ti OR 'trainings, exercise':ab,ti OR 'exercise performance'

#5 'diets, vegetarian':ab,ti OR 'vegetarian diets':ab,ti OR 'vegetarian diet':ab,ti OR 'lacto-vegetarian diet':ab,ti OR 'diet, lacto-vegetarian':ab,ti OR 'diets, lacto-vegetarian':ab,ti OR 'lacto vegetarian diet':ab,ti OR 'lacto-vegetarian diets':ab,ti OR 'plant-based diet':ab,ti OR 'diets, plant-based':ab,ti OR 'plant based diet':ab,ti OR 'plant-based diets':ab,ti OR 'diet, plant-based':ab,ti OR 'diet, plant based':ab,ti OR 'plant-based nutrition':ab,ti OR 'nutrition, plant-based':ab,ti OR 'plant based nutrition':ab,ti OR 'lacto-ovo vegetarian diet':ab,ti OR 'diet, lacto-ovo vegetarian':ab,ti OR 'diets, lacto-ovo vegetarian':ab,ti OR 'lacto ovo vegetarian diet':ab,ti OR 'lacto-ovo vegetarian diets':ab,ti OR 'vegetarian diet, lacto-ovo':ab,ti OR 'vegetarian diets, lacto-ovo':ab,ti OR 'vegetarianism':ab,ti OR 'ovo-vegetarian':ab,ti OR 'diets, vegan':ab,ti OR 'diet, vegan':ab,ti OR 'vegan diets':ab,ti OR 'veganism'

#6 'randomized controlled trial':ab,ti OR 'randomized controlled trials':ab,ti OR 'rct':ab,ti OR 'rcts':ab,ti OR 'cct':ab,ti OR 'ccts':ab,ti OR 'clinical trials':ab,ti OR 'clinical trial'

#7 #1 OR #4

#8 #2 OR #3 OR #5

#9 #7 AND #8AND #6

**Search strategies of** EBSCO

S1(MH "Exercise")

S2(MH "Diet, Vegetarian")

S3 (MH "Diet, Vegan")

S4 SU (Exercises) OR (Physical Activity) OR (Activities, Physical) OR (Activity, Physical) OR (Physical Activities) OR (Exercise, Physical) OR (Exercises, Physical) OR (Physical Exercise) OR (Physical Exercises) OR (Acute Exercise) OR (Acute Exercises) OR (Exercise, Acute) OR (Exercises, Acute) OR (Exercise, Isometric) OR (Exercises, Isometric) OR (Isometric Exercises) OR (Isometric Exercise) OR (Exercise, Aerobic) OR (Aerobic Exercise) OR (Aerobic Exercises) OR (Exercises, Aerobic) OR (Exercise Training) OR (Exercise Performance)

S5 SU (Diets, Vegetarian) OR (Vegetarian Diets) OR (Vegetarian Diet) OR (Lacto-Vegetarian Diet) OR (Diet, Lacto-Vegetarian) OR (Diets, Lacto-Vegetarian) OR (Lacto Vegetarian Diet) OR (Lacto-Vegetarian Diets) OR (Plant-Based Diet) OR (Diets, Plant-Based) OR (Plant Based Diet) OR (Plant-Based Diets) OR (Diet, Plant-Based) OR (Diet, Plant Based) OR (Plant-Based Nutrition) OR (Nutrition, Plant-Based) OR (Plant Based Nutrition) OR (Lacto-Ovo Vegetarian Diet) OR (Diet, Lacto-Ovo Vegetarian) OR (Diets, Lacto-Ovo Vegetarian) OR (Lacto Ovo Vegetarian Diet) OR (Lacto-Ovo Vegetarian Diets) OR (Vegetarian Diet, Lacto-Ovo) OR (Vegetarian Diets, Lacto-Ovo) OR (Vegetarianism) OR (Diets, Vegan) OR (Vegan Diets) OR (Vegan Diet) OR (Veganism)

S6 S1 OR S4

S7 S2 OR S3 OR S5

S8 S6 AND S7

S9 SU Randomized Controlled Trial OR Randomized Controlled Trials OR RCT OR RCTs OR CCT OR CCTs OR Clinical trials OR Clinical trial

S10 S8 AND S9

**Supplementary Table 2: Risk of bias in included Non-RCTs.**

|  | Pischke et al. (2006) | | Dod,et (2010) | | Cairo et al.(2020) | | Slavíček et al.(2008) | | Koeder et al. (2022) |
| --- | --- | --- | --- | --- | --- | --- | --- | --- | --- |
| A clearly stated aim: The question addressed should be precise and relevant in the light of available literature. | “The main aim of the MLDP was to examine whether patients can avoid revascularization by making comprehensive lifestyle changes without increasing cardiac events.” Analysee effect of a plant-based diet and exercise on body compositions. | | We evaluated the influence of the Multisite Cardiac Lifestyle Intervention Program, an ongoing health insurance-covered lifestyle intervention on endothelial function and inflammatory markers of atherosclerosis in this pilot study | | The purpose of this study was to evaluate if a readily available mobile health intervention, ie, Vida, can lead to healthier lifestyle habits for breast cancer survivors. | | The aim of the present study was to measure the parameters of some risk factors before and after a one-week NEW START rehabilitative retreat.The parameters were lower in lacto-ovo vegetarians and Seventh-day Adventists than in controls who never observed the diet and avail the lifestyle programs. | | The objective of the study was to test the effect of a lifestyle intervention on body weight and other chronic disease risk markers. |
| Inclusion of consecutive patients: All patients potentially fit for inclusion (satisfying the criteria for inclusion) have been included in the study during the study period (no exclusion or details about the reasons for exclusion). | We examined medical characteristics, lifestyle, and quality of life by diabetic status and gender in the Multicenter Lifestyle Demonstration Project (MLDP), a study of 440 nonsmoking patients with CAD. Eligibility criteria for study participation have been reported previously. | | Inclusion criteria were (1) age ⬎18 years, (2) mentally competent and able to provide consent, and (3) stable medication and physical health for ⬎4 months. Exclusion criteria were (1) patients unable to finish intensive life style changes and (2) change in dose or new medication started during the study. | | The process of including and excluding participants is not described. | | The process of including and excluding participants is not described. | | The only inclusion criteria were the physical and mental ability to take part in the study (self-reported) and to be ≥18 years old. The process of excluding participants is not described. |
| Prospective collection of data: Data were collected according to a protocol established before the beginning of the study. | The prospective collection of data was not described. | | No description. | | The prospective collection of data was not described. | | The data protocol was maintained for every person in the retreat. | | No description. |
| Endpoints appropriate to the aim of the study: Unambiguous explanation of the criteria used to evaluate the main outcome, which should be in accordance with the question addressed by the study. Also, the endpoints should be assessed on an intention-to-treat basis. | Comparisons of group differences (presence/absence of DM, first-year graduate vs drop-out) in baseline demographic, clinical, risk factor, and psychosocial variables. An intention-to-treat analysis was not mentioned in the study. | | The primary end point of this study was change in flow-mediated dilatation (FMD) after 3 months. The secondary end point was change in the inflammatory, endothelial, and angiogenesis markers after 3 months. An intention-to-treat analysis was not mentioned in the study. | | All the results were recorded to compare changes before and after. | | The questionnaire was completed, then body weight, BMI, blood pressure, heart rate, serum cholesterol and blood glucose were measured. | | Measurements and blood sampling were all performed in the morning and in the fasted state. All the results were recorded to test the study hypotheses. |
| Unbiased assessment of the study endpoint: Blind evaluation of objective endpoints and double-blind evaluation of subjective endpoints. Otherwise the reasons for not blinding should be stated. | Outcomes were analysed by laboratory. Whether blind method is used to extract the results has no influence on the experiment. | | Outcomes were analysed by another laboratory. Whether blind method is used to extract the results has no influence on the experiment. | | Outcomes were analysed by another laboratory. Whether blind method is used to extract the results has no influence on the experiment | | Outcomes were analysed by laboratory. It remains unclear if assessors were blinded. | | Outcomes were analysed by another laboratory. Whether blind method is used to extract the results has no influence on the experiment. |
| Follow-up period appropriate to the aim of the study: The follow-up should be sufficiently long to allow the assessment of the main endpoint and possible adverse events. | Most of the changes were already evident at 3 months and were maintained over 1 year. | | At the beginning of the study and after 3 months FMD in  the brachial artery was assessed in the experimental and control groups according to standard guidelines | | At 6 months, more patients in the app group experienced weight loss and had a significantly greater reduction in overall body mass index (P<0.01). | | The study lasted thirty a-week. | | The duration of follow-up included10 weeks, 6 months, and 1 year. |
| Loss to follow up less than 5%: All patients should be included in the follow-up. Otherwise, the proportion lost to follow-up should not exceed the proportion experiencing the major endpoint. | Drop-out rates in the MLDP ranged from 21% to 27% depending on gender and DM status and compared favorably with those in other follow-up studies with cardiac patients. | | 1 underwent coronary artery bypass surgery, 1 could not follow the exercise program, 1 moved out of town, and 1 could not adhere to the dietary regime)technicians in both the mixed- and LOV-diet groups an accident. | | One study subject passed away (diagnosis not related to cancer) during the study, and 2 others were diagnosed with metastatic disease and did not complete the study. | | The rate of loss to follow-up was less than 5%. | | Voluntarily left study (n = 4);Other missing values (n = 3);Dropouts between 10 weeks  and 1 year (n = 10) - Voluntarily left study (n = 10) |
| Prospective calculation of the study size: Information of the size of detectable difference of interest with a calculation of 95% confidence interval, according to the expected incidence of the outcome event, and information about the level for statistical significance and estimates of power when comparing the outcomes. | Not described. | | Not described. | | Not described. | | The results showed that 6 measured cardiovascular disease risk factor parameters decreased during 30 one-week NEW START retreats in all participating persons (p<0.000l). | Assuming a dropout rate of at least 10%, a minimum sample size of 93 participants (intervention: 62; control: 31) was indicated to reach a global power of 0.8 and a global significance level of 0.05. | |
| An adequate control group: Having a gold standard diagnostic test or therapeutic intervention recognized as the optimal intervention according to the available published data. | Control group patients,who were matched to group 1 patients by procedure eligibility, age, gender, left ventricular ejection fraction, and cardiac score. | | 27 in the experimental group who followed Multisite Cardiac Lifestyle Intervention Program as per standard protocol,4,5 and the remaining 20 historically (age, gender, CAD and CAD risk factors) matched participants were in the control group with usual standard of care. | | The Self group (control arm) received the same printed survivorship information, | | The control group comprised persons who never practised the diet or the lifestyle programmes. | For the control group, a total of 87 participants were recruited (shown in Fig. 1). | |
| Contemporary groups: Control and studied group should be managed during the same time period (no historical comparison). | As subjects served as their own controls, results were not estimated contemporarily. | | As subjects served as their own controls, results were not estimated contemporarily. | | As subjects served as their own controls, results were not estimated contemporarily. | | As subjects served as their own controls, results were not estimated contemporarily. | As subjects served as their own controls, results were not estimated contemporarily. | |
| Baseline equivalence of groups: The groups should be similar regarding the criteria other than the studied endpoints. Absence of confounding factors that could bias the interpretation of the results. | Participants served as their own controls. There was no description about the confounding factors. | | Participants served as their own controls. There was no description about the confounding factors. | | The only variable that was statistically different at baseline was age. | | The measurement of risk factors was not obligatory, but voluntary. This is the reason why many variables were not recorded in all subjects. | Participants served as their own controls. There was no description about the confounding factors. | |
| Adequate statistical analyses: Whether the statistics were in accordance with the type of study with calculation of confidence intervals or relative risk. | | Comparisons of group differences (presence/absence of DM, first-year graduate vs drop-out) in baseline demographic, clinical, risk factor, and psychosocial variables were performed with 2-sample t tests (for continuous variables) and chi-square tests | Baseline characteristics for the 2 groups were compared  using Student’s t and chi-square tests as appropriate. | | | Baseline characteristics are summarized as counts and percentages for categorical variables and means and standard  deviations (SD) for continuous variables and were compared between the 2 groups by methods (t-test and Pearson’s chi-squared). | The statistics: two-sampled t-tests were used for Tables 2 and 3, paired t-tests for Tables 4, and 5. | The statistics: Fisher’s exact test, t test, the independent t test, the Shapiro-Wilk test, a paired t test, a one-way analysis of covariance (ANCOVA), Spearman’s rho correlations (two-sided). | |
| points | | 14 | | 15 | | 11 | 16 | 16 | |

0 - Not reported; 1 - Reported, but inadequate; 2 - Reported and adequate

Red: High risk; Yellow: unclear risk; Green: Low risk.

**Supplementary Table 3: Criteria for defining high, intermediate, and low risk of bias in cohort studies**

Quality assessment of included studies by Newcastle-Ottawa Scale.

| First author, publication (year) | Selection | | | |  | Comparability | |  | Outcome | | | | Score |
| --- | --- | --- | --- | --- | --- | --- | --- | --- | --- | --- | --- | --- | --- |
|  | Representativenes of exposed cohort | Selection of non-exposed cohort | Exposure Ascertainment | Outcome not present at start of study |  | Study controls for diet and exercise | Study controls for any additional important factor |  | Assessment of Outcome | Length of follow-up | Adequacy of follow-up | |  |
| Marshall et al. (2009) |  | ★ | ★ | ★ |  | ★ | ★ |  | ★ | ★ | ★ | 8 | |
| Chainani-Wu et al.(2011) |  | ★ | ★ | ★ |  | ★ | ★ |  | ★ | ★ |  | | 7 |
| Morton et al(2014) | ★ | ★ | ★ | ★ |  | ★ |  |  | ★ | ★ |  | | 7 |
| Null et al. (1996) | ★ | ★ | ★ | ★ |  | ★ | ★‘’ |  | ★ | ★ | ★ | | 9 |
| Kent et al.(2013) | ★ | ★ | ★ | ★ |  | ★ |  |  | ★ | ★ |  | | 7 |
| Kent et al.(2018) |  | ★ |  | ★ |  | ★ | ★ |  | ★ | ★ | ★ | | 7 |
| Diehl et al.(1998) |  | ★ | ★ | ★ |  | ★ |  |  | ★ | ★ | ★ | | 7 |
| Koertge et al.(2003) | ★ | ★ | ★ | ★ |  | ★ |  |  | ★ | ★ | ★ | | 8 |
| Pischke et al. (2007) | ★ | ★ |  | ★ |  | ★ |  |  | ★ | ★ | ★ | | 7 |
| Telles et  al. (2010) | ★ | ★ | ★ | ★ |  | ★ | ★ |  | ★ | ★ | ★ | | 9 |
| Ahrens et al.(2021) |  | ★ | ★ | ★ |  | ★ | ★ |  | ★ | ★ | ★ | | 8 |
| Swi ˛atkiewicz et al. (2021) |  | ★ |  | ★ |  | ★ | ★ |  | ★ | ★ | ★ | | 7 |
| [Suazo](https://pubmed.ncbi.nlm.nih.gov/?size=200&term=Suazo+EMH&cauthor_id=34549028) et al. (2021) | ★ | ★ | ★ | ★ |  | ★ | ★ |  | ★ | ★ | ★ | | 9 |

The scale awards a maximum of 9 stars per study: 4 stars for adequate selection of cohort participants, 2 stars for comparability of cohort participants based on study design and analysis, and 3 stars for adequate determination of outcomes. Studies receiving equal to or more than 7 stars were considered high quality.
