# Supplementary material for: Effects of a vegetarian diet combined with aerobic exercise on glycemic control, insulin resistance, and body composition: a systematic review and meta-analysis
Source: Eat Weight Disord. 2023 Feb 15;28(1):9. doi: 10.1007/s40519-023-01536-5 (PMC9931794; doi:10.1007/s40519-023-01536-5)
Supplement: Supplementary file 2 — Supplementary file2 (DOC 888 KB) [file 40519_2023_1536_MOESM2_ESM.doc]

1. **Forest Figures of Subgroup Analysis**

For BW and BMI, there was an increase in heterogeneity for both BW (SMD: -0.27 95% CI: -0.44, -0.09; *P*=0.003; I²: 88.2%) and BMI (WMD: -0.69; 95% CI: -1.44, 0.06; *P*=0.072; I²: 93.6%) at the intervention ≥ 1 month. In contrast, heterogeneity decreased for both BW (SMD: -0.04; 95% CI: -0.32, 0.23; *P*=0.761; I²: 0%) and BMI (WMD: -0.58; 95% CI: -1.80, 0.63; *P*=0.348; I²: 0%) at the intervention <1 month. However, only the decline in intervention ≥ 1 month was statistically significant (Figure 1and 2).


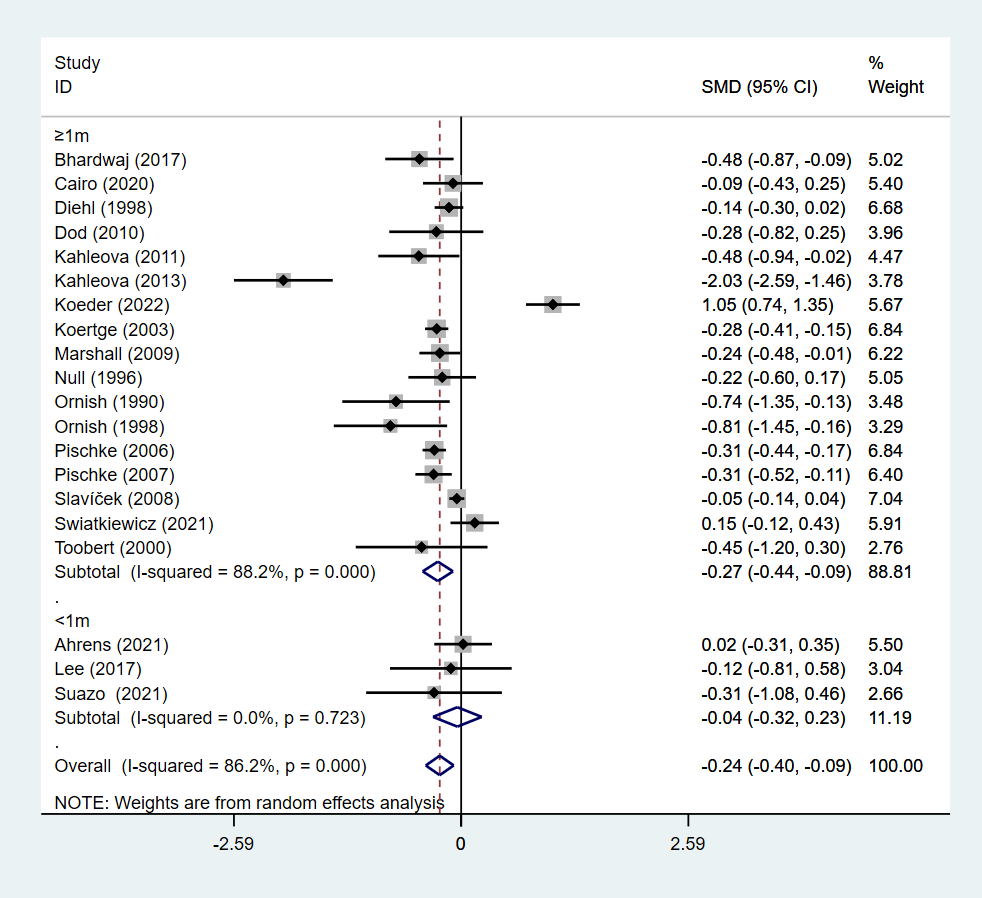


Figure 1 Forest figure of subgroup analysis (BW). For each study, squares represent the mean difference in intervention effects, with horizontal lines intersecting them as the lower and upper limits of the 95% CI. The size of each square represents the relative weight of the studies conducted in the meta-analysis. The diamond represents the results of the meta-analysis combining the individual studies.


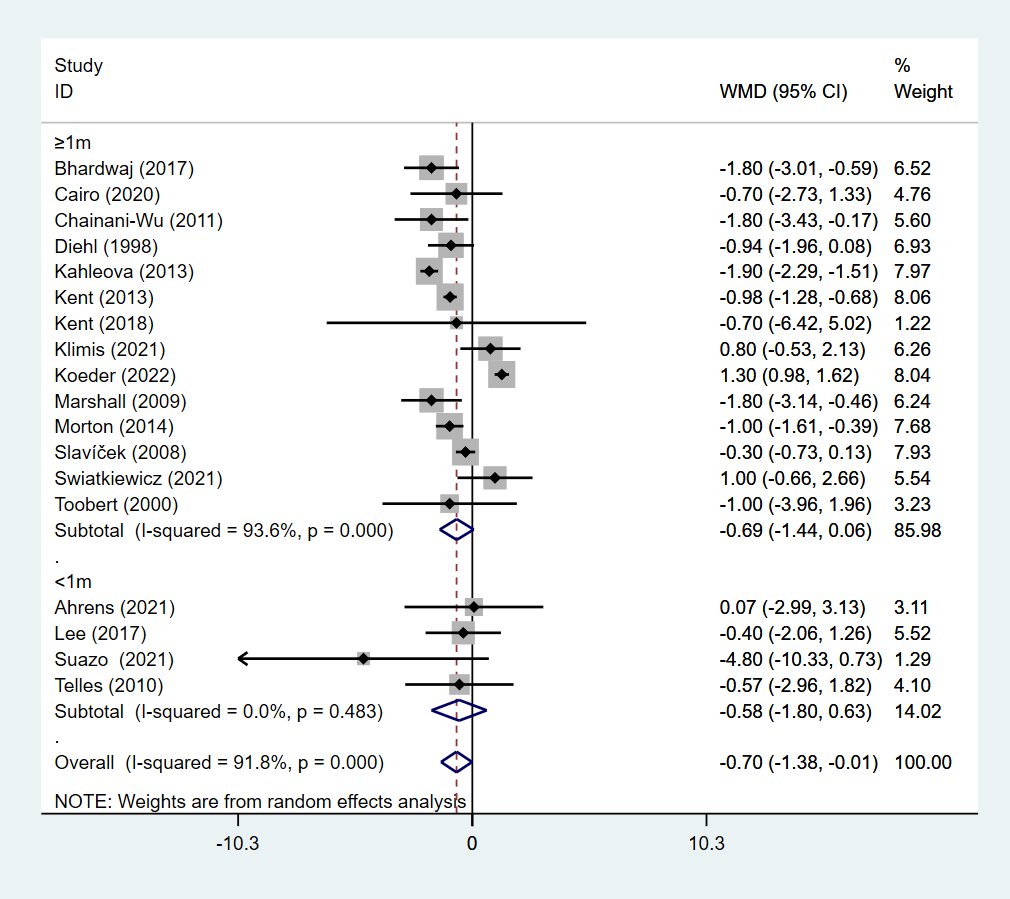


Figure 2 Forest figure of subgroup analysis (BMI). For each study, squares represent the mean difference in intervention effects, with horizontal lines intersecting them as the lower and upper limits of the 95% CI. The size of each square represents the relative weight of the studies conducted in the meta-analysis. The diamond represents the results of the meta-analysis combining the individual studies.

For BF%, there was no way to perform subgroup analysis because there was only one study with an intervention < 1 month. The heterogeneity of BF% (WMD: -1.87; 95% CI: -3.50, -0.24; *P*=0.025; I²: 85.0%) was reduced when the study with an intervention < 1 month was excluded, but neither was statistically significant.

Subgroup analysis of BF% was not possible because only one study was included in the subgroup with an intervention < 1 month.

For WC, heterogeneity decreased at interventions <1 month (WMD: -1.81; 95% CI: -4.01, 0.40; *P*<0.001; I²: 0%) and increased at intervention ≥ 1 month (WMD: -0.81; 95% CI: -5.66, 4.04; *P*=0.744; I²: 95.8%). Substantial heterogeneity was still present even if the intervention was ≥ 1 month, but it was not statistically significant (Figure 3and 4).


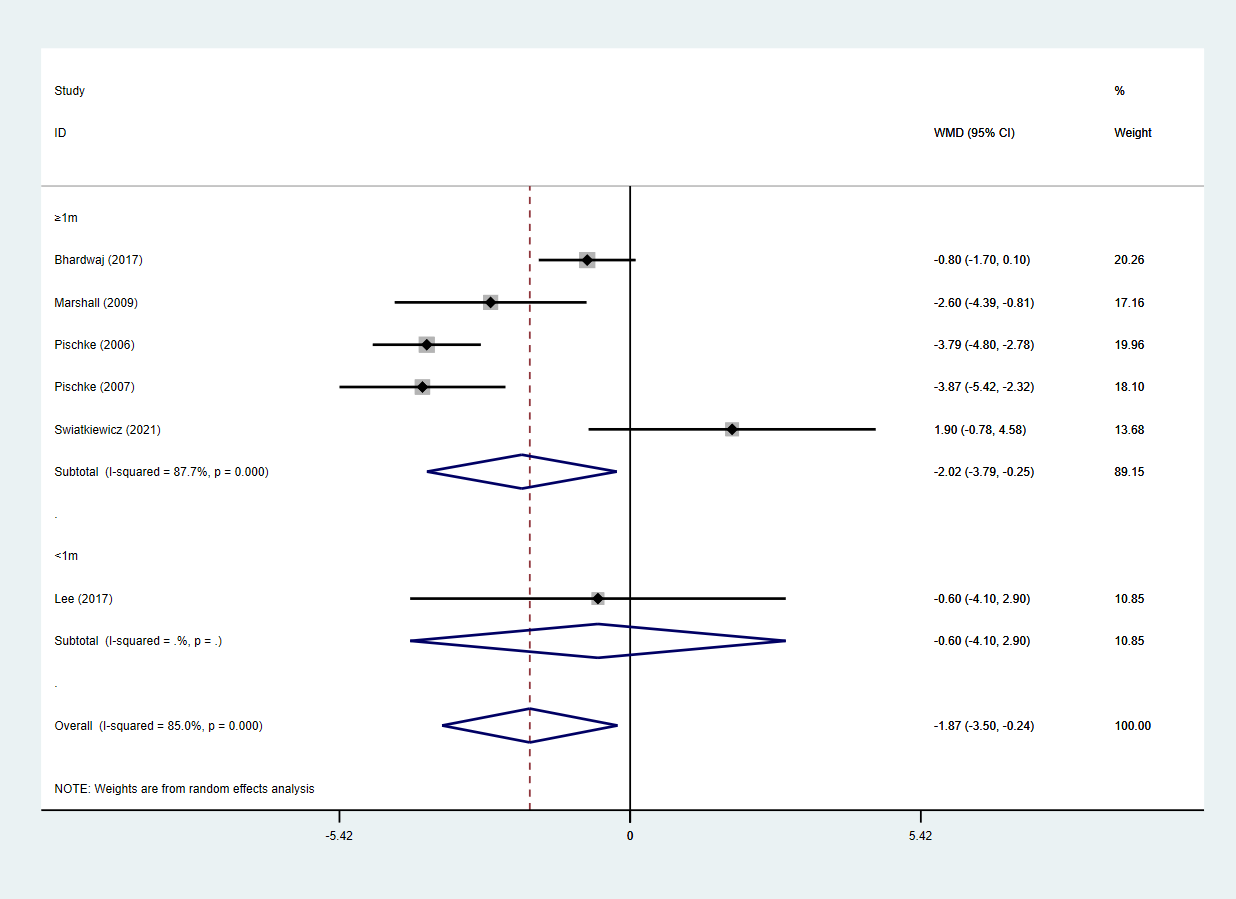


Figure 3 Forest figure of subgroup analysis (BF%). For each study, squares represent the mean difference in intervention effects, with horizontal lines intersecting them as the lower and upper limits of the 95% CI. The size of each square represents the relative weight of the studies conducted in the meta-analysis. The diamond represents the results of the meta-analysis combining the individual studies.


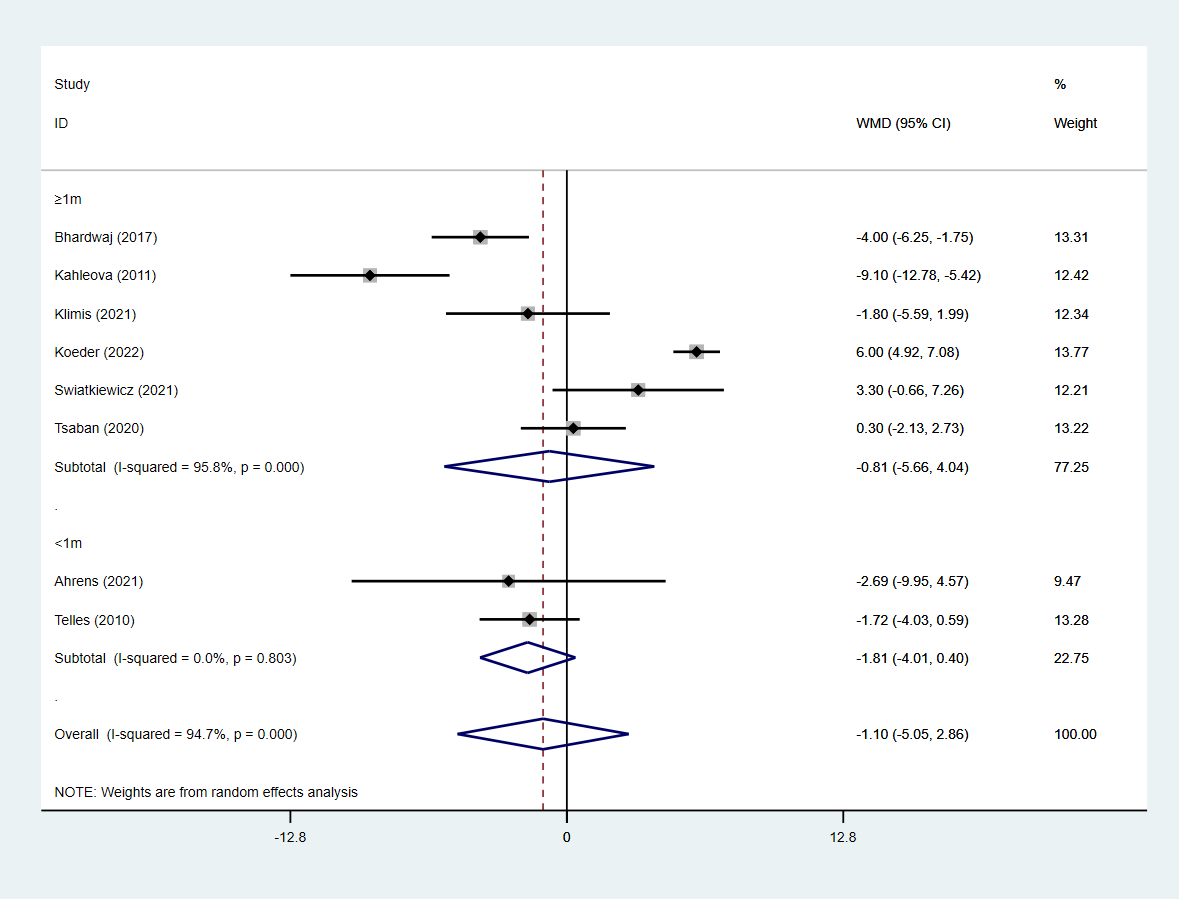


Figure 4 Forest figure of subgroup analysis (WC). For each study, squares represent the mean difference in intervention effects, with horizontal lines intersecting them as the lower and upper limits of the 95% CI. The size of each square represents the relative weight of the studies conducted in the meta-analysis. The diamond represents the results of the meta-analysis combining the individual studies.

1. **Sensitivity Analysis Figures**


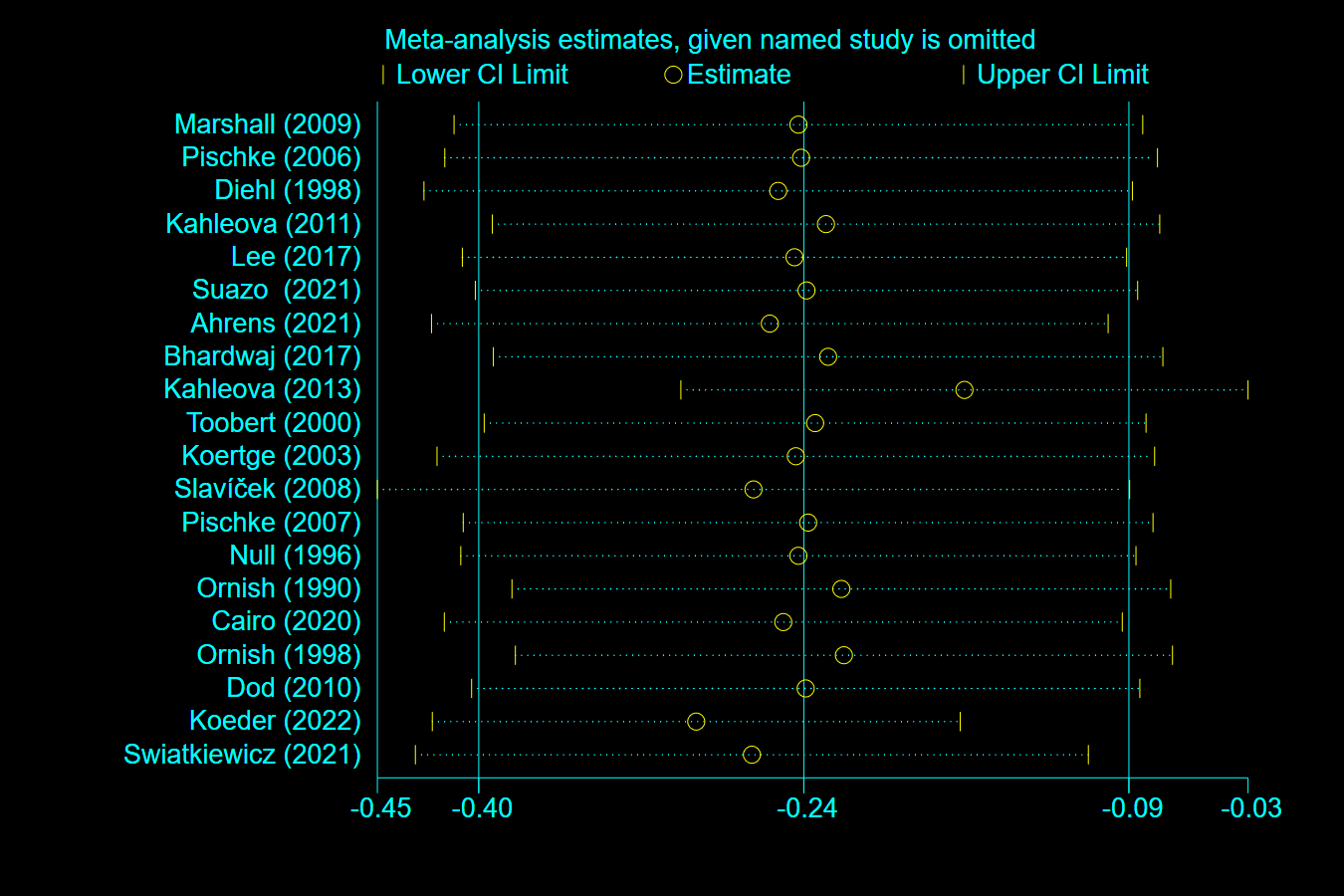


Figure 5 Sensitive Analysis of body weight. Sensitivity analysis was conducted by one-by-one elimination method, and changes in the combined results were observed to evaluate whether the original meta-analysis results had significantly changed under the influence of some studies.


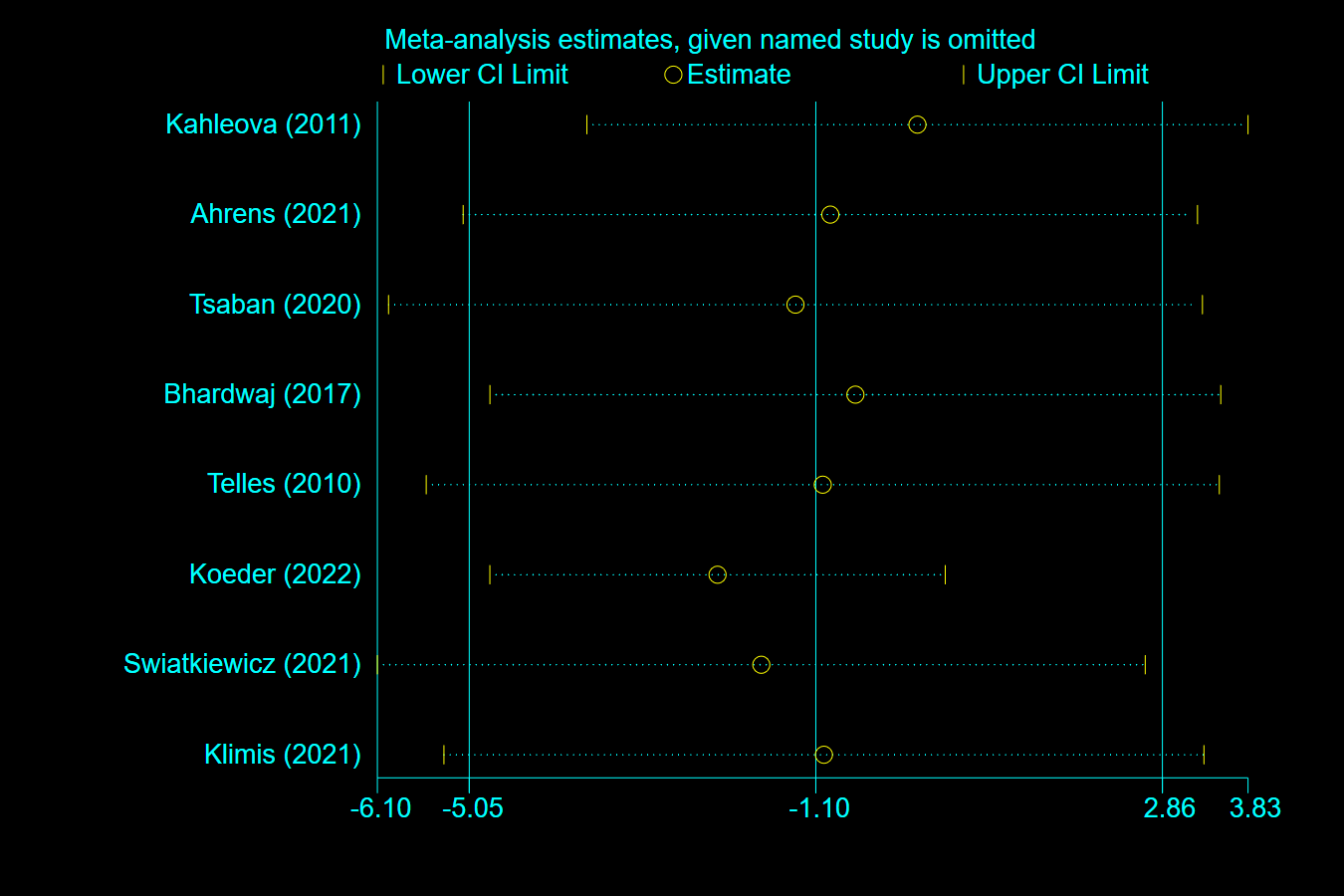


Figure 6 Sensitive Analysis of waist circumference. Sensitivity analysis was conducted by one-by-one elimination method, and changes in the combined results were observed to evaluate whether the original meta-analysis results had significantly changed under the influence of some studies.


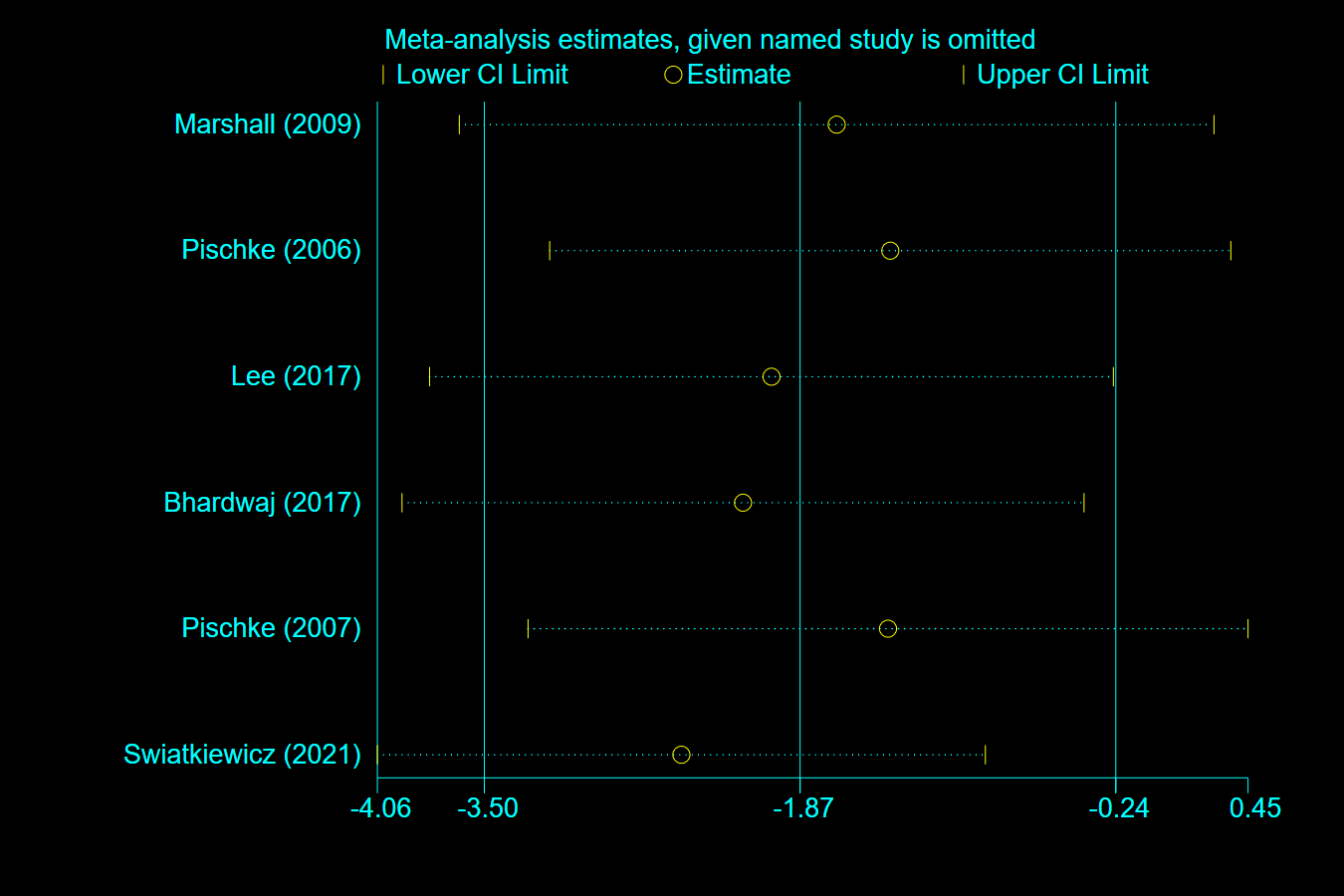


Figure 7 Sensitive Analysis of body fat percentage. Sensitivity analysis was conducted by one-by-one elimination method, and changes in the combined results were observed to evaluate whether the original meta-analysis results had significantly changed under the influence of some studies.

1. **Funnel Plots of Publication bias**


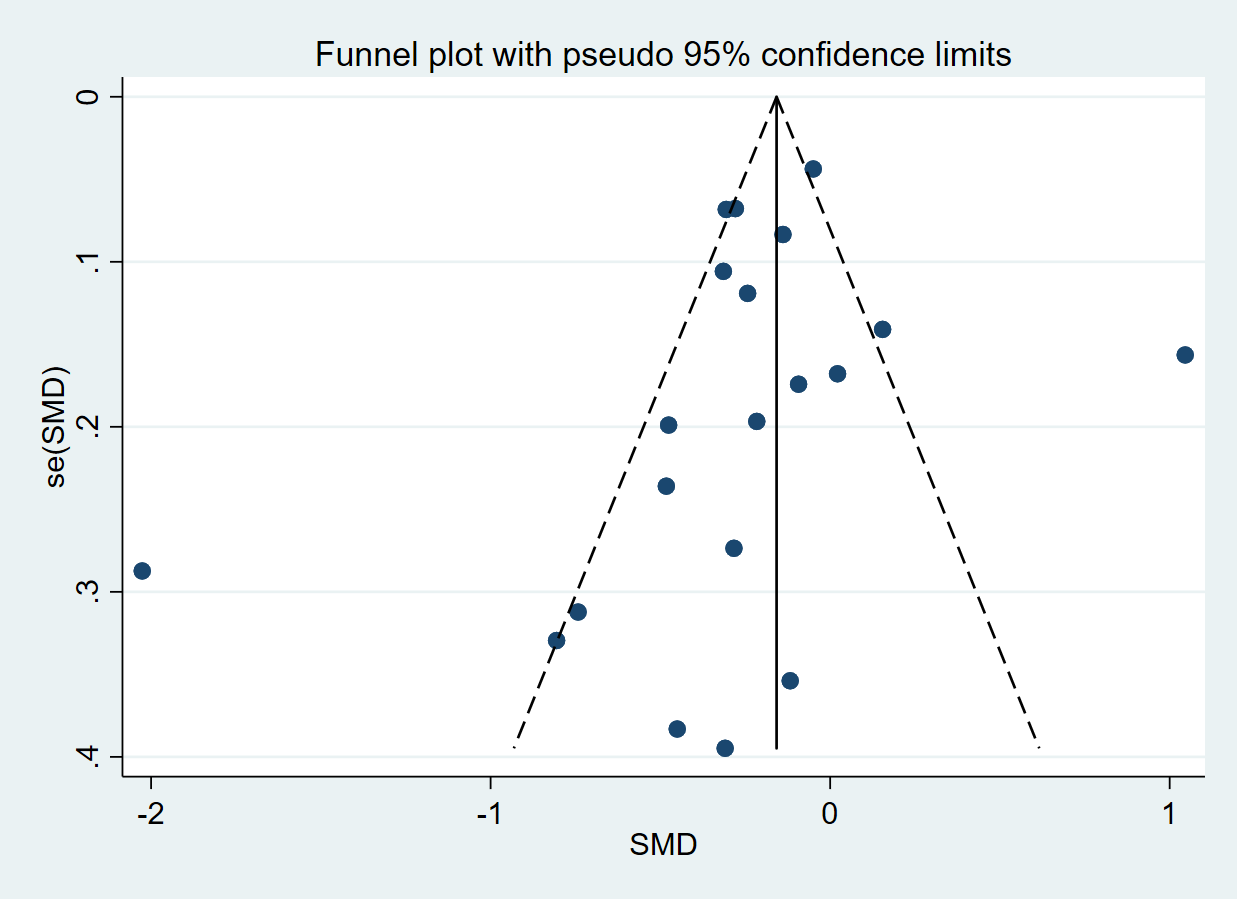


Figure 8 Funnel Plots of body weight. The points are evenly distributed on both sides, indicating no significant publication bias.


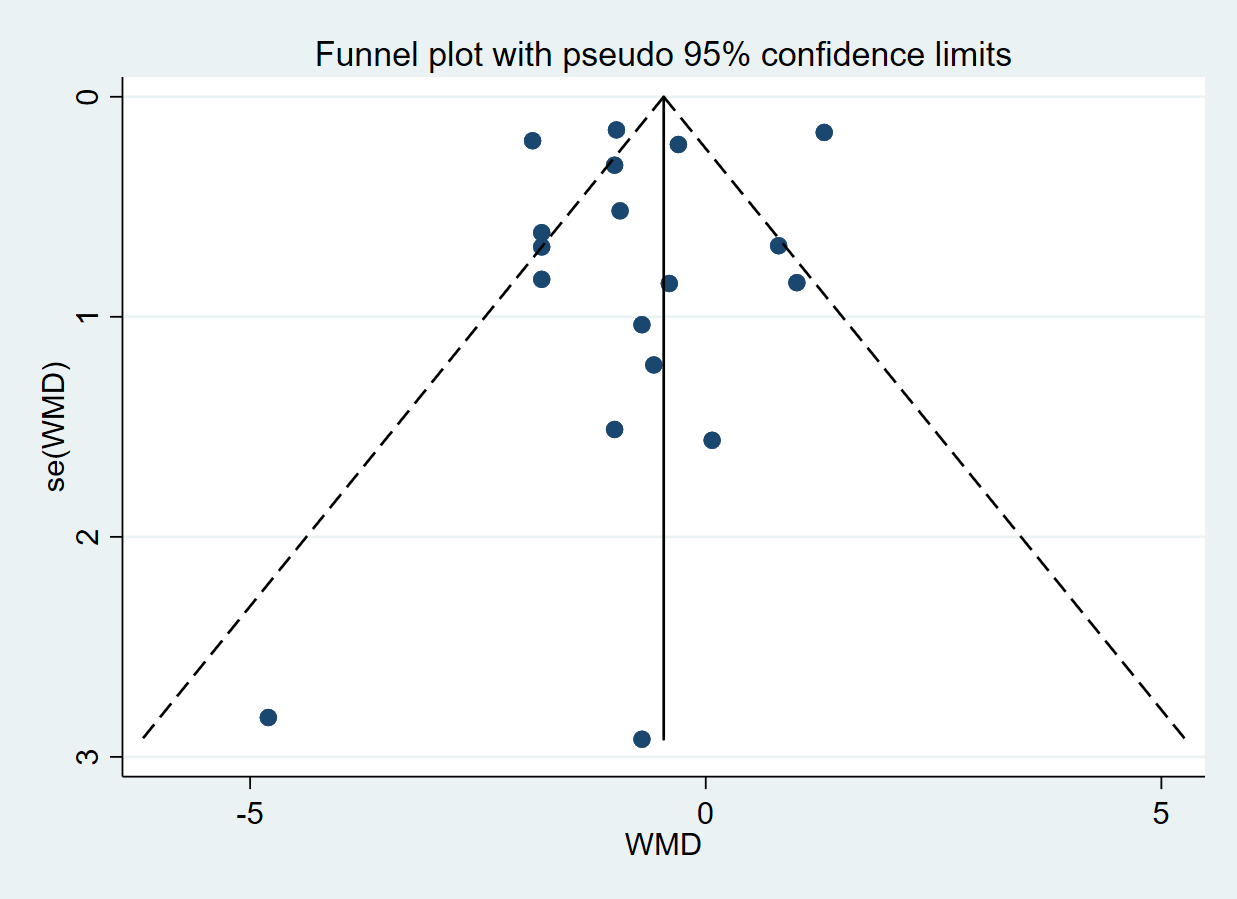


Figure 9 Funnel Plots of Body Mass Index. The points are evenly distributed on both sides, indicating no significant publication bias.


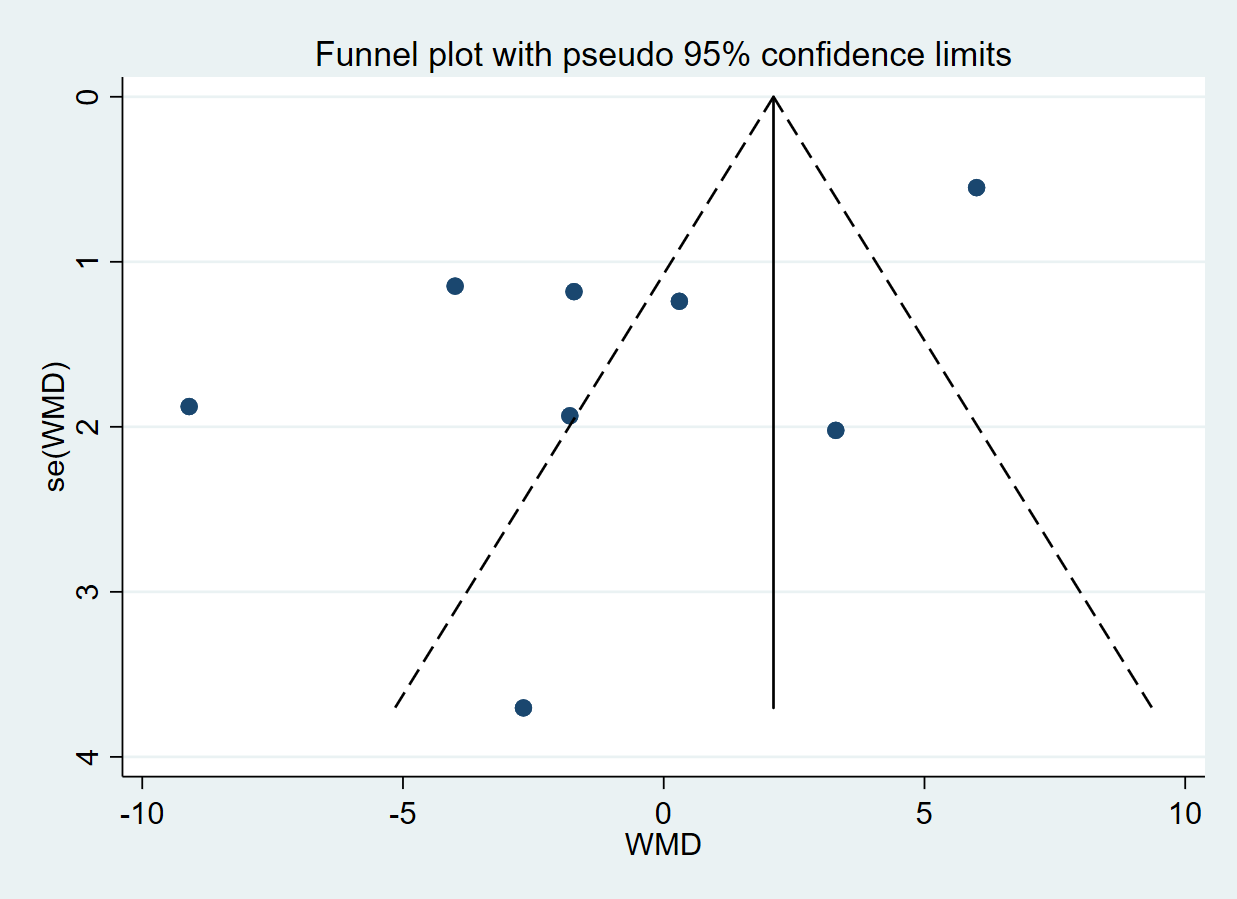


Figure 10 Funnel Plots of waist circumference. Nearly half of the points were distributed outside the border, indicating publication bias.
